# Molecular Mechanisms of Iron Metabolism and Overload

**DOI:** 10.3390/biomedicines13092067

**Published:** 2025-08-25

**Authors:** Aditi Tayal, Jasmeen Kaur, Payam Sadeghi, Robert W. Maitta

**Affiliations:** 1 University Hospitals Cleveland Medical Center, Cleveland, OH 44106, USA; aditi.tayal@uhhospitals.org (A.T.); jasmeen.kaur@uhhospitals.org (J.K.); payam.sadeghi@uhhospitals.org (P.S.); 2 Department of Pathology, Case Western Reserve University School of Medicine, Cleveland, OH 44106, USA

**Keywords:** iron metabolism, iron excess, molecular mechanisms, transfusion, iron overload, metabolism, heme, ferroportin, hepcidin

## Abstract

Iron represents an essential element required for normal physiologic processes throughout organ systems. A vast network of transporters is involved not only in uptake of this element but in processing, oxidation, and recycling to maintain it in a tight balance to avoid excess storage. This complex network of transporters, including heme and ferroportin, among many others, are responsible for facilitating inter-organ tissue iron exchange and availability, contributing to overall heme homeostasis. However, exposure to high levels of iron can overwhelm compensatory mechanisms that result in its accumulation and toxicity. This is the case of patients with genetic diseases such as hemoglobinopathies who suffer from chronic anemia and require, in most instances, a lifetime of red blood cell transfusions to overcome disease crises. Thus, in light of the extensive role of iron in the body, the aim of this review is to present important metabolic pathways involved in iron homeostasis across the cardiovascular, reproductive, hematopoietic, urinary, respiratory, endocrine, and central nervous systems while contrasting these against negative effects caused by iron excess.

## 1. Introduction

Iron metabolism is essential for a wide array of critical cellular and systemic physiological processes. Iron is needed for DNA synthesis, electron and oxygen transport through synthesis of hemoglobin and myoglobin, ATP production, spermatogenesis, and enzymatic reactions needed for the electron transfer chain [1]. Most of the iron in the body is found coupled to hemoglobin (65%), 10% is present in non-hemoglobin components (myoglobin, enzymes and cytochromes), with the remainder in iron stores across the body [2]. Under normal conditions there is a daily loss of 1–2 mg of iron from the body via shedding of the gastrointestinal lining and skin or through red cell senescence [3]. However, these losses are balanced through careful iron absorption and recycling [4].

There can be at times exposure to increased iron concentrations resulting from either acute blood loss or from exposure to blood transfusions such as patients who are chronically transfused [5]. Physiologic iron balance is regulated by proteins known as iron regulatory proteins (IRPs) which control both its concentration and functions [6,7]. Of these, IRPs 1 and 2, are essential to iron homeostasis at the cellular level, first through binding to iron response elements (IREs) and second to iron translator regions of mRNA-encoding proteins involved in iron uptake, storage, and export via proteins like transferrin receptor 1 (TFR1), divalent metal transporter-1 (DMT1), and ferroproteins [3]. Thus, IRPs bind to IREs when iron concentration in the body is low and dissociate from them when concentration is high [4].

**Figure 1:**
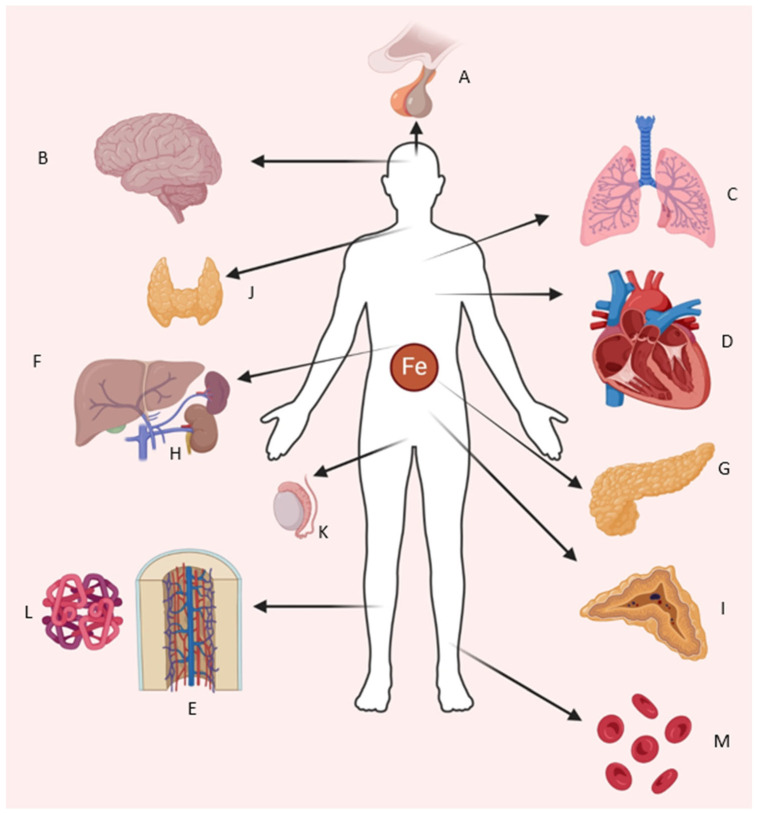
Organ systems requiring iron for normal function and affected by iron overload. Shown are (A) pituitary–hypothalamic axis, (B) central nervous system, (C) lungs, (D) heart and cardiovascular system, (E) bone marrow, (F) liver, (G) pancreas, (H) kidneys, (I) adrenal glands, (J) thyroid gland, (K) testes/reproductive system, (L,M) heme and erythrocytes (Illustration by authors).

A close balance in iron concentration is necessary for proper homeostasis, since iron is toxic when present in excess (overload) and toxicity leads to a variety of human health issues encountered frequently in patient populations exposed to frequent transfusions or among those with a genetic predisposition, such as individuals with hereditary hemochromatosis, sickle cell disease (SCD) and thalassemia [5,7]. Therefore, the aim of this review is to present the latest advances in understanding the molecular regulation of iron metabolism, highlighting its role in cellular homeostasis and overall integration of iron-dependent systemic and cellular processes (Figure 1). The role of iron will be examined looking at essential regulatory networks, exploring pathological consequences of iron dysregulation and excess, while discussing its impact on specific organ systems. This comprehensive approach aims to establish a foundational understanding of iron metabolism across different physiological and pathological contexts, while presenting available therapeutic options once conditions of overload develop.

## 2. Physiologic Absorption of Iron

### 2.1. Absorption

Dietary iron exists in two forms: heme and nonheme [8]. Both forms can be found in plant or animal sources and have distinct absorption rates closely regulated by hepcidin [9]. There are two states in which iron can be found: one is ferrous (Fe^2+^) and the second is ferric (Fe^3+^) [10]. Nonheme iron is predominantly sourced from cereals, vegetables, legumes, and fruits, where it is present in the ferric (Fe^3+^) state. Nonheme iron absorption involves a multi-step process that requires it to be reduced to the ferrous (Fe^2+^) state before absorption occurs [11]. In contrast, heme iron, which is more abundant from animal-based sources, is absorbed more efficiently than the nonheme form. A critical early step in the absorption of nonheme iron requires secretion of hydrochloric acid by the stomach [12]. This form of iron is solubilized and reduced by gastric acid, greatly facilitated by ascorbic acid and other ferric reductases, which precedes its absorption by the small intestine [13]. Gastric acid also preserves ascorbic acid in its active reduced state so that it chelates soluble iron, preventing its precipitation. However, since humans lack the ability to synthesize ascorbic acid, this must be obtained through dietary sources [12].

**Figure 2:**
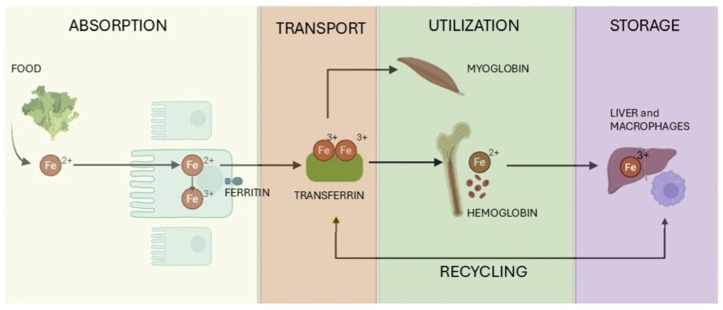
Overview of iron metabolism: Illustration showing key processes in iron metabolism, including intestinal absorption of dietary iron, transport via transferrin, utilization in erythropoiesis and cellular functions, storage in ferritin within the liver and macrophages, and recycling of iron via reticuloendothelial system. (Adapted and modified by authors from: “Iron in the Diet: Iron-Rich Foods, Excess and Deficiency Symptoms.” https://madebydiet.com/eu/iron-in-the-diet-iron-rich-foods-excess-and-deficiency-symptoms/, accessed on 1 April 2025).

Gastric acid in the proximal duodenum reduces Fe^3+^ via membrane-bound ferric reductase duodenal cytochrome B1 (DCYTB) [14]. Iron is then transported across the apical brush border membrane of intestinal epithelial cells by DMT1, also known as SLC11A2, capable of transporting several divalent cations including Fe^2+^, or less commonly via SLC36A1 when bound to nicotianamine [15]. Ferrous iron can subsequently be stored as ferritin or trafficked as needed to the basolateral surface [14]. On this surface, Fe^2+^ is transported to the blood by the iron transporter ferroportin (FPN), a transmembrane domain protein encoded by the *SLC40A1* gene in a process coupled with H^+^ exchange, and hephaestin, an iron regulatory protein that oxidizes iron to its Fe^3+^ state [15,16]. This is why use of proton pump inhibitors which impair gastric acid production results in substantial reduction of iron absorption [2]. Finally, Fe^3+^ binds to transferrin (TF), a liver-derived serum protein, for systemic distribution across the body [17]. Notably, the most important checkpoint for iron absorption is the proximal small bowel which regulates whole-body iron levels [18]. As a result, the interplay between intake and bioavailability establishes how this element is not only needed by the organism, but how its concentration is balanced to prevent both deficiency and toxicity (Figure 2).

### 2.2. Hepcidin and Other Related Mediators

The *HAMP* gene encodes for hepcidin, a 25 amino acid protein synthesized by hepatocytes and to a lesser extent by the intestine and heart, that increases iron availability [19,20]. Hepcidin regulates iron absorption by binding to FPN, in this way blocking iron transport while inducing FPN internalization followed by lysosomal degradation [21,22]. Once internalized, FPN undergoes proteasomal degradation facilitated by the E3 ubiquitin ligase RNF217, the E1 enzyme UBA6, and the adaptor protein NDFIP1 [23]. Importantly, through this binding hepcidin regulates FPN and provides an additional layer of iron flow regulation from enterocytes into circulation [22]. This explains how *HAMP* mutations result in iron overload, as the absence of hepcidin permits constitutively high iron absorption and overall unregulated iron transport. Consequently, hepcidin-mediated FPN downregulation increases enterocyte iron levels, promoting degradation of hypoxia-inducible factor (HIF)-2, which reduces expression of key iron absorption genes such as *DMT1* and *DCYTB* [24]. Hepcidin, like FPN, is itself tightly regulated to maintain iron homeostasis [25]. Its expression increases with iron overload and inflammation but decreases under states of hypoxia or heightened iron demand, such as during increased erythropoiesis. This regulation is mediated by bone morphogenetic protein (BMP)-dependent signaling pathways, primarily involving BMP2 and BMP6 produced by liver sinusoidal endothelial cells [26]. The latter regulates hepcidin transcription and its overall expression [27]. Notably, anemias have also been classified as being characterized by either high or low hepcidin [28]. When hepcidin is persistently high, further iron absorption is halted leading most commonly to iron deficiency anemia; however, under iron excess/overload low hepcidin is more likely observed [29]. This is a tightly regulated feedback mechanism that when impaired leads to those health problems encountered by chronically transfused patients and resulting in higher iron burden.

Hepatocyte surface proteins including hereditary hemochromatosis protein (HFE), hemojuvelin (HJV), transferrin receptor (TFR)2, BMP receptors (ALK2, ALK3, BMPR-II), neogenin, and transmembrane protease serine 6 (TMPRSS6), modulate hepcidin expression in response to BMPs and diferric TF [30]. Of interest, inherited mutations in *HFE*, *HJV*, and *TFR2* cause hereditary hemochromatosis, a condition of iron overload that physiologically impacts multiple organs such as liver, pancreas, heart, and endocrine glands. As a result, hepcidin expression is tigjtly regulated by several mechanisms: BMP signaling via SMAD1/5/8 transcription factors [31], nuclear-related factor-2 (NRF2) activation under iron-induced oxidative stress [32], and erythropoietic activity mediated by erythroferrone [33].

**Figure 3:**
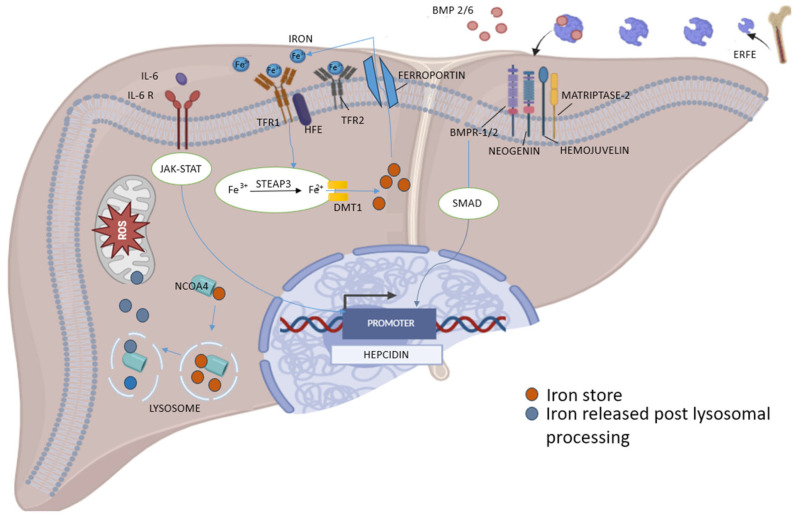
Molecular pathways regulating hepcidin transcription and iron metabolism in liver. Hepcidin expression is regulated by hepatic iron stores and plasma iron levels through the BMP2/6–BMPR–SMAD signaling pathway, modulated by hemojuvelin, matriptase-2, and neogenin. Plasma iron stimulates hepcidin via TFR1–HFE–TFR2 complex enhancing BMP signaling; while inflammation upregulates hepcidin through IL-6 activation of the JAK–STAT pathway. In contrast, erythropoietin-stimulated erythroblasts suppress hepcidin by secreting erythroferrone, which inhibits BMP2/6 signaling. Cellular iron homeostasis involves iron uptake (TFR1, STEAP3, DMT1), export (ferroportin), and storage in ferritin. Autophagic degradation of ferritin (ferritinophagy), mediated by NCOA4, releases iron into the labile pool, increasing reactive oxygen species (ROS) and promoting ferroptosis (Illustration by authors).

Inflammation and inflammatory cytokines, mainly interleukin (IL)-6, also lead to increased levels of hepcidin [34]. This is because IL-6 binding to its receptor and through activation of the JAK/STAT3 signaling pathway increases hepcidin production (Figure 3). These high levels of hepcidin block iron release by FPN, lowering iron concentration and making it less available in circulation [35]. On the contrary, during conditions of increased circulatory iron presence or cases of overload due to high iron exposure, as those seen in inflammatory states and hemoglobinopathies, the net result is decreased absorption of iron by FPN, resulting in slow and gradual restoration of iron homeostasis [36].

The gut microbiome also contributes to regulation of dietary iron absorption and uptake. Intestinal microbes produce metabolites that inhibit iron absorption, thereby ensuring an adequate supply for their own metabolism [37]. For instance, 1,3-diaminopropane, derived from both bacterial and dietary sources, suppresses iron transport proteins DCYTB, DMT1, and FPN by inhibiting HIF-2 activity in enterocytes [37]. These metabolites also enhance ferritin expression, sequestering iron within enterocytes, while reducing its availability and export to the bloodstream. Ultimately, iron stored in ferritin is lost when enterocytes are shed, returning it to the gut microenvironment.

### 2.3. Cellular Nonheme Iron Uptake

Erythroid precursors predominantly acquire circulating diferric TF for erythropoiesis through the TF cycle. In this process, diferric TF binds to TFR1 and is internalized via endocytosis [38]. Endosomal acidification then releases Fe^3+^, which is reduced by the six-transmembrane epithelial antigen of the prostate (STEAP)3 and transported into the cytoplasm by DMT1 (Figure 3) [38]. TFR1 also interacts with serum ferritin, suggesting the existence of an alternative TF-independent iron uptake pathway [39]. Thus, TF, remains central to our understanding of iron homeostasis. Its two iron-binding sites are functionally distinct, as demonstrated by differing binding sensitivities to erythropoietin (EPO) in mice with mutations capable of preventing iron loading in either site [40].

Cellular iron uptake is regulated by IRPs, which control the translation of key mRNAs, including those for TFR1, DMT1, FPN, and ferritin [38]. IRPs bind to IREs in mRNA untranslated regions (UTR), stabilizing or inhibiting their translation depending on the binding site; IRP binding to the 3′ UTR enhances mRNA stability and translation; while binding to the 5′ UTR suppresses it. Of note, the RNA-binding protein Roquin plays a regulatory role by destabilizing TFR1 mRNA [41]. Among IRPs, IRP1 activity is modulated by iron–sulfur (Fe-S) clusters, binding RNA only when these clusters are absent, while IRP2 is regulated through degradation by the FBXL5 E3 ligase in an iron-dependent manner [38]. However, cellular iron import is not limited to the TF cycle. For instance, during iron overload, TF saturation results in enhanced circulation of non-transferrin-bound iron (NTBI), a potentially toxic form of iron [38]. NTBI uptake by liver and pancreas relies on SLC39A14, also known as ZRT/IRT-like (ZIP)14, a protein that primarily transports manganese but that facilitates iron transport during iron excess or overload [38].

### 2.4. Non Cellular Heme Intracellular Iron Trafficking, Utilization, Storage, and Recycling

A substantial portion of cellular iron is directed toward the mitochondria, where it is either stored or utilized for heme and Fe-S cluster synthesis [42]. Iron that is not directed to mitochondria for heme synthesis is stored as Fe^3+^ coupled to ferritin (Figure 2) [38]. Research has implicated multiple pathways in mitochondrial iron uptake, including through direct endosome–mitochondria contact (kiss-and-run model) and acquisition from the labile iron pool (LIP) that comprises redox-active and low-molecular-weight iron species [43]. Another essential mediator in mitochondria iron regulation are the Fe-S clusters. Biogenesis of these clusters is a complex, multistep process involving sulfur donors, iron supply, and specialized proteins that facilitate cluster synthesis, transport, and incorporation into target proteins [44]. This process occurs in both mitochondria and the cytoplasm. These clusters are critical for electron transfer in the respiratory chain and function as cofactors in DNA metabolism, oxygen sensing, and other essential cellular processes. Mutations in key components of this pathway, such as heat shock cognate B, which transfers clusters to target proteins, are linked to diseases like congenital sideroblastic anemia [45]. Impaired lysosomal acidification can also result in disruption of cellular iron uptake, depletion of Fe-S clusters, limit mitochondrial function, and reduce overall cell viability [46]. Notably, defects in Fe-S cluster biosynthesis, such as those associated with frataxin mutations in Friedreich’s ataxia, can be mitigated during hypoxic conditions, as evidenced by improved outcomes in mice exposed to 11% oxygen [47].

Iron that is not used in cellular processes or directed to mitochondria is stored in ferritin. This protein is a hetero-polymer composed of 24 subunits of light and heavy chains, of which the latter has ferroxidase (converts Fe^2+^ to Fe^3+^) activity [48]. Ferritin’s heavy chains exhibit this ferroxidase activity, enabling iron storage in its Fe^3+^ state [38]. The delivery of iron to ferritin is mediated by poly(rC)-binding protein 1 (PCBP1), a multifunctional protein with distinct RNA- and iron-binding capabilities that facilitates iron delivery to other iron-dependent proteins [49]. Particularly, liver-specific deletion of *Pcbp1* in mice results in disrupted oxidative stress, lipid peroxidation, and steatosis, highlighting its critical role in mitigating cytoplasmic iron toxicity [50]. Along these lines, a recent study revealed that exosomal release of ferritin-rich vesicles is regulated by IRP and involves the extracellular vesicle marker CD63 [51]. One of ferritin’s most important functions is mitigating oxidative damage by reducing the pool of free iron available for reactive oxygen species (ROS) generation. Free excess iron catalyzes Fenton reactions which enhance production of hydroxyl radicals that damage lipids, proteins, and DNA. Thus, by storing iron as Fe^3+^, a safe, non-reactive form, ferritin minimizes oxidative stress and protects hepatocytes from ROS-related cellular injury [50,52].

Iron mobilization from ferritin is regulated by “ferritinophagy”, a selective autophagy process carried out by auto-phagosomes and driven by NCOA4 (Figure 3) [53]. Expression of NCOA4 is upregulated during iron deficiency via hypoxia-inducible factors (HIFs) and downregulated under iron excess through proteasomal degradation [53,54]. Thus, in states of iron excess/overload, iron binding sites on ferritin are saturated resulting in decreased iron binding and increased iron delivery to mitochondria [53]. NCOA4 deficiency in mice results in ferritin and iron accumulation in tissues, reduced serum iron levels, and anemia. Tissue-specific deficiencies highlight its role in erythropoiesis and hepatocyte iron mobilization during blood loss [52]. In vitro, NCOA4 deficiency disrupts mitochondrial function, further emphasizing its importance in iron metabolism [52]. Consequently, imbalances in these regulatory systems increase the risk of cell death such as during infection and malignancies, where higher ferroptosis (a form of iron-dependent cell death) provides iron rapidly to proliferating cells [55].

#### 2.4.1. Mitochondria and Fe-S Clusters

Fe-S clusters are cofactors necessary for electron transfer through complexes I, II, and III leading to cytochrome c reduction in the mitochondrial respiratory pathway [56]. Mitochondrial aconitase (ACO2) plays a crucial role in both iron metabolism and cellular energy production within the mitochondria. It is a tricarboxylic acid (TCA) cycle enzyme that mediates conversion of citrate to isocitrate contributing to ATP and heme biosynthesis [56]. ACO2 contains a [4Fe–4S] cluster sensitive to oxidative stress with activity that can be influenced by iron levels and redox signaling, which works as an iron-sensing regulator of both mitochondrial respiration and erythropoiesis [57]. However, ACO2 is highly susceptible to inactivation by ROS, leading to the formation of a [3Fe–4S] cluster resulting in enzyme dysfunction and release of free iron [58]. ACO2 inactivation not only disrupts mitochondrial metabolism but also contributes to the generation of hydroxyl radicals through Fenton-type reactions, exacerbating intracellular oxidative stress [59]. This metabolic shift underscores the interconnectedness of mitochondrial Fe-S clusters, ROS production, and cellular energy metabolism [60].

Under iron-deficient/limiting conditions, ACO2 expression decreases, resulting in reduced mitochondrial citrate levels [61], as well as isocitrate [57]. This decrease does not necessarily impair the overall capacity of the TCA cycle, but indicates a shift in metabolic fluxes for the cell to adapt to lower iron availability [61]. The cytosolic isoform of ACO2 is a known IRP1, playing a major role in cellular iron metabolism by acting as a sensor regulating uptake and storage [62]. In iron-replete conditions, IRP1 catalyzes conversion of citrate to isocitrate [63]. In low iron conditions, the [4Fe–4S] cluster within IRP1 disassembles, converting into an RNA-binding protein that regulates iron homeostasis by binding to IREs found in mRNA of target genes, in this way preventing translation of ferritin and stabilizing TFR1’s mRNA, thus facilitating iron uptake [63]. However, by modulating TFR1 and ferritin expression, IRP1 indirectly influences the efficiency of iron absorption at the cellular level. This regulatory mechanism ensures that cells adapt to changing iron availability, maintaining iron homeostasis and supporting vital functions such as erythropoiesis and mitochondrial energy production [62]. Further discussion of mitochondrial Fe-S clusters will be presented in the context of each organ system in the rest of the manuscript.

#### 2.4.2. Mitochondrial Iron Dysregulation

Mitochondria, as sites for Fe-S cluster biosynthesis and energy generation via oxidative phosphorylation, can be affected by iron excess/dysregulation. Excessive mitochondrial iron leads to elevated ROS levels, mitochondrial membrane depolarization, and impairments in ATP production [64]. Mitochondrial proteins such as mitoferrin (responsible for mitochondrial iron import) exhibit dysregulated expression in neurodegenerative diseases, further disturbing cellular iron balance [64,65]. Since the role of mitochondria in ferroptosis is increasingly recognized, strategies targeting mitochondrial iron dyshomeostasis, such as using antioxidants like MitoTEMPO have shown protective effects in preclinical models of Alzheimer’s and Parkinson’s [66,67].

### 2.5. Iron Elimination and Export

Iron excretion occurs mostly via passive, unregulated pathways, such as the shedding of intestinal and skin epithelial cells, menstruation, and minor/limited epithelial trauma. Recent studies have shed light on additional mechanisms of iron excretion using a TF-deficient mouse model, in which TF treatment promoted gastrointestinal iron excretion, leading to normalization of iron levels [68]. Additionally, hepatic uptake of NTBI via SLC39A14 was identified as a prerequisite for biliary excretion of ferritin-bound iron, though its role in overall iron homeostasis remains unclear [69]. Paradoxically, a study of anemic children exposed to stable iron isotopes revealed increased iron losses during iron supplementation, potentially linked to occult gastrointestinal bleeding; however, the definitive pathways mediating this phenomenon require future research [70].

FPN (SLC40A1) is critical for exporting iron from cells involved in storage and recycling such as enterocytes and macrophages [71]. It transports Fe^2+^ into the bloodstream, where it is oxidized to Fe^3+^ by ferroxidases like hephaestin and ceruloplasmin, enabling TF-mediated systemic transport [72]. As mentioned previously, FPN’s role in iron homeostasis makes it a primary target of hepcidin regulation. This regulatory step adjusts plasma iron levels in response to systemic signals during iron sufficiency, deficiency, inflammation, or increased erythropoiesis. Elevated hepcidin during iron sufficiency or inflammation inhibits FPN, reducing iron efflux and limiting plasma iron availability; conversely, low hepcidin levels during iron deficiency or heightened erythropoietic activity allow FPN to remain active, promoting iron mobilization for essential functions such as hemoglobin synthesis [73]. In hereditary hemochromatosis, hepcidin production is reduced, causing excessive FPN activity and systemic iron overload that results in oxidative stress, liver fibrosis, cirrhosis, and potentially hepatocellular carcinoma [74]. On the other hand, chronic inflammation elevates hepcidin levels, suppresses FPN activity, and traps iron within macrophages [75]. As a result, this functional iron deficiency contributes to anemia despite adequate iron stores.

In addition to systemic regulation by circulating hepcidin, FPN-mediated iron export is also influenced by intracellular and local environmental signals that help fine-tune iron elimination at the cellular level. These hepcidin-independent mechanisms enable cells to respond to their own iron needs and local physiological conditions, allowing for more precise control of iron handling beyond endocrine signaling. In duodenal enterocytes, FPN expression is regulated at both the translational and transcriptional levels [71]. The IRE–IRP system suppresses FPN translation during intracellular iron deficiency; however, enterocytes express an alternative FPN mRNA isoform that lacks the 5′ IRE, allowing continued translation even when iron is scarce [71]. This ensures ongoing iron export into the bloodstream, even under local deficiency. Additionally, in states of hypoxia or systemic iron deficiency, the transcription factor HIF-2α is stabilized and upregulates FPN, DMT1, and DCYTB [76]. This promotes both apical iron absorption from the intestinal lumen and basolateral iron export into circulation.

In macrophages, especially those involved in iron recycling from senescent erythrocytes, FPN expression is modulated by intracellular signals such as heme and iron levels. During red blood cell (RBC) breakdown, excess heme inactivates the repressor BACH1, allowing the transcription factor NRF2 to bind antioxidant response elements (AREs) and stimulate FPN transcription, facilitating safe iron export [77]. Oxidative stress from high intracellular iron further activates NRF2, enhancing FPN expression and protecting against iron-induced toxicity [77]. In contrast, during inflammatory states, activation of Toll-like receptors (TLRs) by microbial products suppress FPN mRNA expression, thereby reducing iron export [78]. This contributes to hypoferremia, an innate immune response limiting iron availability to pathogens. Furthermore, microRNAs (miRNAs)—including miR-485-3p, miR-20a, and miR-20b—have been implicated in post-transcriptional FPN downregulation in both enterocytes and macrophages [79]. While the physiological significance of these miRNAs remains to be fully understood, they represent an additional layer of control in fine-tuning iron export under specific cellular settings.

### 2.6. Pathways Modulating Hepcidin

#### 2.6.1. Erythropoietin-Responsive Factor Erythroferrone (ERFE)

The renal medulla, which under physiologic conditions consumes significant energy, monitors oxygen delivery by monitoring hemoglobin levels, oxygen binding, and oxygen release [33]. When oxygen delivery falls short of demand, interstitial fibroblasts detect hypoxia and produce EPO [33], and its production is closely regulated by HIF-2 [80]. In response to EPO, erythroid precursors in the bone marrow divide and mature into erythrocytes, a process requiring iron for heme and hemoglobin synthesis. Thus, iron deficiency disrupts heme and hemoglobin production and by default impairs erythrocyte development [81]. At baseline, erythropoiesis consumes most circulating plasma iron [28]. When erythropoiesis is stimulated—by hypoxia, blood loss, or exogenous EPO—it suppresses hepcidin which enables increased iron absorption from the diet and mobilization from stores to meet physiologic demand for heme and hemoglobin production [82].

However, EPO does not directly suppress hepcidin but instead uses an intermediary EPO-responsive factor ERFE to this effect [83]. Once plasma iron levels and liver iron stores increase, hepcidin synthesis is stimulated by the activation of the SMAD1/5/8 pathway [84]. This activation is driven by BMPs, especially the BMP2/6 heterodimer, through binding of the BMP receptor (R) complex [84,85]. ERFE effectively suppresses this pathway via matripase-2, a serine protease encoded by the *TMPRSS6* gene, which cleaves the hepcidin activator HJV [86]. However, this mechanism has been disputed by studies using *TMPRSS6*^−/−^ mice, which mimic iron-refractory iron deficiency anemia, showing that elevated BMP-SMAD signaling leads to increased hepcidin production despite increased ERFE levels [87]. Specifically, disruption of ERFE showed minimal impact on their hematological phenotype or hepcidin production. Furthermore, in vitro studies of hepatocytes from *TMPRSS6*^−/−^ mice have indicated that ERFE suppresses hepcidin expression independently of TMPRSS6, even under BMP-stimulated conditions [87]. This may be due to ERFE lowering hepcidin levels via binding of BMP ligands and inhibition from interacting with the cell surface receptor ALK3 [88].

##### ERFE Pathophysiology—Baseline Erythropoiesis and Stress Erythropoiesis

Under normal conditions, the bone marrow constantly produces RBCs to replace senescent cells or damaged ones, with most of their iron content recycled by macrophages in the spleen and liver. When anemia or low oxygen levels occur, renal cells sense the reduced oxygen supply and respond by increasing EPO production through HIF-2 signaling [89,90]. This higher EPO synthesis enhances the survival of RBC precursors, boosting their numbers and prompting these precursors to secrete ERFE [90]. ERFE then lowers hepcidin production in the liver resulting in increased FPN activity, allowing cells to release additional iron into the bloodstream [91]. Subsequently, dietary iron absorption rises, and stored iron from macrophages and hepatocytes becomes available for hemoglobin production in new RBCs. ERFE is especially critical early in the response to increased RBC demands as shown in *ERFE*-deficient mice, in which hepcidin suppression after blood loss or EPO treatment is delayed, thus slowing anemia recovery by several days compared to wild type animals [91]. Therefore, when anemia develops, ERFE production increases for two main reasons: first, EPO stimulation causes the pool of erythroid precursor cells to expand, and second, each of these precursors increases ERFE production [92]. However, during ineffective erythropoiesis, although there are far more erythroid precursors driven by high EPO levels, most of them fail to mature into healthy RBCs. Instead, these “stalled” precursors release large amounts of ERFE, persistently lowering hepcidin and leading to iron overload [92]. This excess iron that does not bind to TF can then generate harmful ROS as seen in β-thalassemia patients [93]. This iron-driven toxicity subsequently damages cells particularly sensitive to high iron levels such as liver, heart, and endocrine glands, while simultaneously raising the infection risk [92].

##### ERFE Variants

Excessive ERFE levels play an important role in iron-loading anemias. Variations in the *ERFE* gene alter the protein’s activity, affect how long ERFE mRNA or protein persists, or both [94]. For example, a point mutation in the C1q domain (A260S) leads to higher ERFE RNA and protein levels than normal, even in healthy individuals [94]. This same mutation has been found in some patients with congenital dyserythropoietic anemia type II, who have markedly increased ERFE levels due to ineffective RBC production [95]. In these patients, the mutation pushes ERFE RNA and protein levels even higher, causing more severe anemia [95]. This increased ERFE worsens the condition by raising iron availability for erythropoiesis; however, in doing so it boosts oxidative stress in a background of already dysfunctional erythroblasts.

##### ERFE as a Biomarker in Chronic Kidney Disease

Chronic kidney disease (CKD) often leads to anemia due to both lower EPO levels and limited iron availability [96]. This anemia has several causes, including inflammation, reduced EPO production, and poor hepcidin clearance by the damaged kidneys. Current treatment guidelines from expert panels recommend regularly checking RBC counts and iron levels, and supplementing iron and EPO if patients become deficient [97,98]. Interestingly, an animal model of CKD has shown that removing hepcidin improved anemia, suggesting that in patients whose anemia is mainly due to high hepcidin levels, future therapies that mimic or boost ERFE might prove therapeutic [96]. Although giving EPO does increase ERFE naturally, it happens more slowly in CKD mice compared to wild type [99]. This delay reduces EPO’s effectiveness at releasing iron, indicating that directly supplementing ERFE might help mobilize iron and improve anemia in CKD.

##### Myelodysplastic Syndromes and ERFE

Myelodysplastic syndromes (MDS) are blood disorders where immature blood cells fail to develop properly, often dying in the bone marrow. Over time, surviving abnormal cells gain additional mutations and progress toward overt leukemia. A subtype called MDS-RS (with ring sideroblasts) features immature RBCs loaded with excess iron in their mitochondria, preventing them from properly maturing and entering the bloodstream [100]. Many MDS patients—especially those with MDS-RS—have mutations in splicing factor genes, most notably *SF3B1* [100,101]. MDS-RS patients with *SF3B1* mutations develop systemic iron overload, even without receiving transfusions. The presence of mutated ERFE transcripts in cells with *SF3B1* mutations encoding an ERFE protein variant with four extra amino acids, can still suppress hepcidin as effectively as native ERFE [102]. These mutated cells synthesize more ERFE than normal cells, and this higher production is linked to improved cell survival in *SF3B1*-mutant MDS patients. Thus, since ERFE is produced in erythroblasts, measuring it could prove useful in managing MDS-RS [102].

##### Beta-Thalassemia and ERFE

Animal models of anemias involving ineffective erythropoiesis generally exhibit markedly elevated ERFE levels, because the number of ERFE-producing erythroblasts expands beyond what would be expected given the degree of anemia. For example, the *Hbb^(th3/+)^* mouse—manifesting a non–transfusion-dependent form of β-thalassemia—displays moderately reduced hemoglobin levels, significantly lower hepcidin during growth, and elevated plasma and liver iron concentrations [103]. This arises from β-globin haploinsufficiency that leads to buildup of unpaired α-globin chains. In these mice, ERFE expression in bone marrow and spleen remains consistently high (8- to 32-fold above wild-type) across all ages studied [104]. However, use of ERFE-specific neutralizing antibodies alleviates anemia in this model. This indicates that excess ERFE contributes to both iron overload and development of anemia [104,105]. Notably, antibody treatment also reduces the reticulocyte percentage, implying a possible improvement in RBC quality and lifespan using this approach. However, it remains to be determined whether ERFE inhibition in humans with β-thalassemia can sufficiently reduce iron absorption and deposition to eliminate the need for concurrent iron-chelation therapies.

#### 2.6.2. BMP Signaling via SMAD1/5/8 Transcription Factors

The earlier mentioned BMP-SMAD pathway regulates hepcidin, and this modulates iron levels through its binding of FPN, inducing both its internalization and degradation. This process restricts iron absorption from the intestine and limits its release from cellular stores. This signaling pathway orchestrates the transcriptional regulation of the *HAMP* gene, in response to systemic iron levels and other regulatory feedback signals [106]. It should become evident by now that hepcidin, as the master regulator of systemic iron homeostasis, is controlled by multiple pathways converging on its transcriptional and post-transcriptional regulation to maintain a balance between iron availability and storage. Consequently, its expression is transcriptionally regulated through a feedback loop that allows hepatocytes to sense circulating iron levels. This regulatory feedback depends on several proteins on hepatocytes’ plasma membrane, including HFE, TFR2, and HJV, which collectively modulate hepcidin synthesis via the BMP6 signaling pathway [106]. BMP6, a member of the TGF-β superfamily, is produced in hepatocytes, with its expression upregulated by increased liver iron concentrations [107]. BMP6 binds to BMPR and HJV, initiating an intracellular signaling cascade mediated by SMAD proteins [107]. Likewise, even though BMP6 is the primary driver stimulating hepcidin expression, phosphorylated SMAD1, SMAD5, and SMAD8 form complexes with SMAD4, which then translocate to the nucleus to activate *HAMP* [108]. On the other hand, hepatocytes’ TFR1 and TFR2, along with HFE, function as iron sensors that monitor TF-bound iron (TF-Fe). In this way, under low iron conditions HFE binds to TFR1, and when iron levels rise and TF-Fe saturates, TFR1 and HFE are displaced [108]. The freed HFE then associates with TFR2, forming an HFE/TFR2 complex that interacts with HJV to activate the BMP/SMAD pathway and promote hepcidin production. Disruption of this regulatory mechanism, such as HFE deficiency, results in hereditary hemochromatosis [108].

BMP/SMAD signaling pathway is involved in the suppression of hepcidin expression by epidermal growth factor (EGF) [109]. EGF downregulates hepcidin by reducing SMAD1/5/8 phosphorylation, a key downstream step in BMP receptor (BMPR) activation, thereby interfering with BMP-mediated transcription of *HAMP* [31]. This effect is BMP6-dependent, as EGF did not suppress hepcidin in mice lacking BMP6, and was reversed by BMP6 supplementation, confirming the necessity of BMPR-SMAD signaling in EGF-mediated hepcidin regulation [106]. SMAD1/5/8 signaling also plays a functional protective role in liver injury and fibrosis during iron overload. Mice with hepatocyte-specific deletion of *Smad1*, *Smad5*, and *Smad8* exhibited markedly increased hepatic iron accumulation, reduced hepcidin expression, and significant signs of liver pathology, including fibrosis and inflammation when fed a high-iron diet [31]. This lends further support to the critical role for the BMP-SMAD1/5/8 axis in regulating systemic iron levels via hepcidin and protecting the liver from iron-induced injury and fibrotic remodeling.

#### 2.6.3. NRF2 Activation Under Iron-Induced Oxidative Stress

Under normal conditions, the transcription factor NRF2 is bound to Keap1, a protein that mediates its ubiquitination and proteasomal degradation [110]. Oxidative stress, especially from iron-induced ROS, modifies cysteine residues on Keap1, preventing NRF2 degradation and thus stabilizing it; this subsequently translocates to the nucleus, where it binds to AREs in the promoter regions of target genes [111]. These genes encode antioxidant enzymes such as heme oxygenase-1 (HO-1), NADPH quinone oxidoreductase 1, and glutamate-cysteine ligase catalytic subunit, among others [111]. NRF2 also regulates and promotes expression of ferritin and FPN [112]. These actions decrease the LIP, mitigating ROS production and maintaining redox homeostasis. While NRF2 activation protects cells from oxidative damage, its hyperactivation in cancer cells contributes to tumor progression [113]. Malignant cells, characterized by high iron demands for proliferation and metabolism, produce excessive ROS due to increased mitochondrial activity [113]. Hyperactive NRF2 counteracts ROS, supporting cancer cell survival, proliferation, and resistance to chemotherapy and radiotherapy [111]. NRF2 also facilitates cancer cell adaptation to ferroptosis driven by lipid peroxidation and iron accumulation [114]. Thus, by inducing antioxidant defenses and regulating iron export, NRF2 diminishes ferroptosis sensitivity, offering a survival advantage to cancer cells [114]. As a result, inhibition of NRF2 can restore ferroptosis sensitivity, presenting a potential therapeutic strategy in malignancies.

#### 2.6.4. IL-6 Pathway

Inflammatory cytokine IL-6 plays a pivotal role in triggering hepcidin production through the IL-6 receptor (IL-6R)/STAT3 signaling pathway [115]. During infections involving iron-dependent pathogens, inflammatory cytokines such as IL-6 are generated as part of the innate immune response, a process primarily mediated by macrophages, which release IL-6 in response to infection/inflammation. The increased IL-6 levels activate STAT3 signaling, leading to a rise in hepcidin production and subsequent iron sequestration [116]. The IL-6R/STAT3 pathway is crucial in immune responses, inflammation, iron homeostasis, and the acute-phase response [117]. The pathway begins with IL-6 binding to its receptor, either membrane-bound or soluble [117]. This IL-6/IL-6R complex associates with gp130 (a transmembrane protein that works as a shared cytokine coreceptor), forming a hexameric receptor complex that triggers intracellular signaling. Subsequent activation of Janus kinases (JAK1 and JAK2) associated with gp130 leads to phosphorylation of tyrosine residues, creating docking sites for the transcription factor STAT3 [116]. This factor is phosphorylated, dimerized, and translocated to the nucleus, where it binds to specific DNA sequences known as STAT3-responsive elements on the promoters of target genes [118]. In this way, STAT3 regulates transcription of genes involved in hepcidin production, acute-phase proteins, and cellular processes such as survival, proliferation, and differentiation [116]. IL-6-induced STAT3 signaling upregulates hepcidin expression, sequestering iron to limit its availability to pathogens during infections while enhancing immune responses by promoting production of inflammatory mediators and acute-phase reactants [117]. Dysregulation of the IL-6R/STAT3 pathway is implicated in chronic inflammatory diseases, autoimmune disorders such as rheumatoid arthritis, and malignancies [119]. Therapeutic targeting of this pathway, such as with IL-6R inhibitors may prove effective in managing these conditions.

#### 2.6.5. ZIP14

ZIP14, a member of the ZRT/IRT-like protein family, is the primary hepatic transporter for NTBI. Located on the basolateral membrane of hepatocytes, ZIP14 facilitates NTBI uptake during iron overload, aided by its metal-binding residues and structure comprising of eight transmembrane domains [120]. NTBI internalization depends on prior iron reduction mediated by surface ferrireductases such as DCYTB and STEAP, and prion protein (PrP^D^) [121]. ZIP14 is essential for protecting extrahepatic tissues from iron toxicity by sequestering NTBI in the liver, as shown in hereditary hemochromatosis [122]. In such cases, dysregulated hepcidin and FPN activity result in excessive iron absorption, TF saturation, and NTBI in plasma, which ZIP14 removes [120]. However, prolonged hepatic iron accumulation due to ZIP14 activity contributes to fibrosis, cirrhosis, and hepatocellular carcinoma if left untreated [123]. Regulation of ZIP14 expression by inflammatory cytokines like IL-6 couples increases in iron uptake to infection and chronic inflammation [122]. ZIP14 localization is also upregulated in response to ferric ammonium citrate [120]. In contrast, DMT1 is needed for iron acquisition during deficiency, with protein levels increasing by 200% under iron-deficient conditions [124]. DMT1 expression dominates in the pancreas, where its mRNA levels are 3.8 times higher than ZIP14, supporting its role in the TF cycle [125]. Together, these transporters enable the liver to maintain systemic iron balance: ZIP14 handles NTBI during overload, while DMT1 supports TF-Fe iron uptake during deficiency. This underscores their therapeutic potential for addressing iron-related disorders such as hemochromatosis and iron-deficiency anemia [125].

#### 2.6.6. Prion (PrP^D^) Protein

PrP^D^ appears to play an important function in hepatic iron uptake and storage. PrP^D^ facilitates hepatic cellular absorption of both TF-Fe and NTBI through its ferrireductase activity [126,127]. Experiments with *PrP^D−/−^* mice revealed that absence of PrP^D^ significantly impairs NTBI uptake and iron storage, as shown by reduced NTBI uptake and lower Fe-ferritin levels [127]. Additionally, PrP^D^ enhances iron transport by upregulating ZIP14 and DMT1 activity, while both ZIP14 and DMT1 modulate PrP^D^ processing, decrease full-length PrP^D^ and increase truncated forms, through mechanisms involving PrP^D^ recycling or selective cleavage [127]. Further research is needed to establish how this protein is integrated in overall hepatic iron regulation.

## 3. Bone Marrow and Heme Iron

Heme iron bioavailability is enhanced by the alkaline pH of the small intestine, which prevents polymerization. While nonheme iron uptake mechanisms are well-characterized, heme iron absorption remains less understood but likely involves two potential mechanisms: receptor-mediated endocytosis and membrane transport [128]. Historically, the heme receptor was described as a high-affinity, pH-dependent heme-binding protein in the small intestine with activity influenced by trypsin digestion [129]. Iron deficiency enhances heme uptake and binding by duodenal cells [130]. Localization studies have revealed heme accumulation at microvilli and in endosomal compartments of duodenal cells [131]. Heme carrier protein 1, now known as the proton-coupled folate transporter (SLC46A1), was originally misidentified as an intestinal heme importer but is primarily a high-affinity, pH-dependent folate transporter [132]. Mutations in *SLC46A1* lead to hereditary folate malabsorption, which is treatable with folate supplementation and does not affect iron metabolism. However, the mechanism of intestinal heme transport remains unclear due to lack of appropriate genetic models, as mice are inefficient heme absorbers [133]. In this context, the heme reporter HRG1—a four-transmembrane-domain heme transporter known for its role in macrophage iron recycling—has emerged as a promising candidate for intestinal heme absorption. HRG1 is expressed in the human small intestine facilitating heme import through endocytic compartments, suggesting a role in intracellular heme trafficking. Support for this function comes from studies in *Caenorhabditis elegans*, a heme auxotroph that relies entirely on environmental heme uptake [134]. Through genome-wide microarray analysis, researchers identified *hrg1* and *hrg4* as genes upregulated during heme deficiency [135]. Both genes encode transmembrane proteins with distinct localizations and functions: HRG4 is located at the plasma membrane and mediates heme uptake from the intestinal lumen, while HRG1 localizes to intracellular vesicles such as endosomes and lysosomes, where it regulates intracellular heme distribution [135]. Functional conservation of HRG1 was confirmed in mammals, where its expression facilitates heme transport across membranes [136], and in human small intestine HRG1 facilitates heme import through endocytic compartments [137]. These findings highlight HRG1 as a therapeutic target for conditions related to dysregulated heme metabolism or parasitic infections dependent on host-derived heme.

### 3.1. Heme Synthesis

Heme is an iron-containing tetra-pyrrole that is important in binding oxygen, globin transportation, and detoxification [14]. Heme’s functions include activation of transcription factors BACH1 and NRF2, GATA 1-mediated gene expression, as antioxidant during cellular stress, and as regulator of cell proliferation and apoptosis through activation of these transcription factors [138]. Heme also regulates the circadian rhythm and cell cycles similar to mitochondrial respiration and Fe-S clusters [139]. Hydrophobic heme is cytotoxic due to generation of ROS, which in turn results in enhanced oxidative stress responses [140]. Because of this, heme homeostasis is tightly regulated at synthesis, import, utilization, degradation, and export [141].

The synthesis of 5-aminolevulinate (ALA) from succinyl-CoA and glycine, catalyzed by ALA synthase (ALAS), is the first and rate-limiting step of heme biosynthesis occurring in the mitochondrial matrix [142]. ALAS exists in two isoforms: ALAS1, expressed ubiquitously, and ALAS2, specific to erythroid cells. While heme tightly regulates ALAS1 at multiple levels, ALAS2 is not subject to heme-mediated regulation [142]. Heme regulates ALAS1 by destabilizing its mRNA, promoting degradation, and inhibiting both its maturation and mitochondrial import [143]. After its synthesis, ALA exits the mitochondria via the *SLC25A38* gene product, though this is also involved in glycine transport and Fe-S cluster biogenesis [144,145]. In the cytosol, two ALA molecules are enzymatically combined by ALA dehydratase to form porphobilinogen, but this enzyme requires an Fe-S cluster for function [146]. Four porphobilinogen units are subsequently assembled into hydroxymethylbilane by hydroxymethylbilane synthase, which is first converted into uroporphyrinogen III by uroporphyrinogen synthase, followed by decarboxylation by uroporphyrinogen decarboxylase to form coproporphyrinogen III, which re-enters the mitochondria [147]. There, the latter is sequentially converted first into protoporphyrinogen IX and later to protoporphyrin IX (PPIX) by enzymes coproporphyrinogen oxidase and protoporphyrinogen oxidase, respectively. Finally, ferrochelatase (FECH) catalyzes insertion of Fe^2+^ into PPIX to produce heme on the inner mitochondrial membrane [147]. Iron delivery to FECH is mediated by mitoferrin 1 and 2; and FECH interacts with ALAS and protoporphyrinogen oxidase, as well as mitochondrial α-ketoglutarate dehydrogenase, succinyl-CoA synthase, and the porphyrinogen transporter TMEM14C [148]. Additionally, FECH forms complexes with mitoferrin, ABC transporters ABCB7 and ABCB10, and heme chaperones PGRMC1 and PGRMC2 [149].

#### 3.1.1. Protoporphyrin IX

PPIX requires eight molecules of glycine (from plasma) and eight molecules of succinyl-CoA to form the tetrapyrrole macrocycle [150]. The plasma membrane glycine transporter 1 and mitochondrial transporter SLC25A38 are required for normal erythropoiesis [151]. Deficiencies in either transporter has a negative impact on heme synthesis and results in anemia; specifically, mutations in the *SLC25A38* gene are responsible for inherited recessive sideroblastic anemia [145,151,152].

#### 3.1.2. Posttranscriptional Modifications

Phosphorylation, lysine acylation, and cysteine glutathionylation are key to regulating metabolic pathways and their disruption can result in disease states [153,154,155]. The [2Fe-2S] cluster in FECH is sensitive to the cell’s acidity (pH) and membrane charge, meaning that it adjusts FECH’s activity based on the cell’s energy state and environment, and in this way regulates heme synthesis by coupling it to the cell’s overall energy and redox balance [156].

#### 3.1.3. Dysfunctional Heme Synthesis

Unlike iron deficiency anemia, which primarily arises from insufficient iron for heme synthesis, anemia of chronic disease (ACD)/inflammation involves dysfunctional heme synthesis among additional factors [157]. One such factor is the impact of inflammation over erythropoiesis, mediated by macrophages in erythroblastic islands that interact closely with developing erythroid cells [157]. During inflammation, macrophages upregulate the *Irg1* gene, which encodes aconitate decarboxylase responsible for catalyzing the production of itaconate, an antimicrobial compound that disrupts the TCA cycle by inhibiting succinate dehydrogenase, leading to increased succinate [158,159,160]. While itaconate is not synthesized by erythroid cells, it is actively transported into them to be converted to itaconyl-CoA via succinyl-CoA:glutaryl-CoA transferase, which exchanges succinate in succinyl-CoA with itaconate [161]. This process reduces cellular succinyl-CoA, a critical substrate for ALAS2, the first enzyme in the heme synthesis pathway [161]. Likewise, itaconyl-CoA directly inhibits ALAS2 as a competitive inhibitor, hereby reducing production of ALA, the initial heme precursor. Itaconyl-CoA similarly inhibits ketoglutarate dehydrogenase, in this way limiting succinyl-CoA availability. This duality of itaconate—depleting succinyl-CoA and inhibiting ALAS2—reduces hemoglobin synthesis during inflammation [161]. This resembles hepcidin, in its inhibition of heme synthesis by regulating iron [162]. Together, itaconate and hepcidin act as complementary mechanisms to limit the production of potentially toxic intermediates (iron and protoporphyrin) during inflammation, tightly regulating heme synthesis in ACD. ALAS2 is itself regulated at both translational and post-translational levels. Under low iron conditions, apo-IRP1 binds to the 5′ UTR of ALAS2 mRNA inhibiting its translation; while when iron is plentiful, holo-IRP1 (containing an Fe-Su cluster) acts as a cytosolic aconitase interconverting citrate and isocitrate [163]. Post-translationally, the mitochondrial protease complex CLPXP—consisting of the unfoldase CLPX and the protease CLPP—governs ALAS2 turnover, and in erythroid cells CLPX not only regulates ALAS2 levels but influences other terminal heme synthesis enzymes (protoporphyrinogen oxidase and FECH) as well as mitochondrial iron metabolism [164].

### 3.2. Heme in Erythrocytes

In human bone marrow and fetal liver, hematopoietic stem cells sequentially generate burst-forming and colony-forming erythroid progenitors, then proerythroblasts, followed by basophilic, polychromatic, and orthochromatic erythroblasts that give origin to 2.5 billion erythrocytes per second [165]. Late-stage erythroblasts undergo enucleation and organelle loss to form reticulocytes, which enter the bloodstream and mature into RBCs [166]. The transition from proerythroblast to erythroblast occurs in specialized “erythroblastic islands,” where a central nurse macrophage supports a surrounding ring of RBC precursors, a process during which developing erythrocytes demand high levels of iron to fuel heme and hemoglobin synthesis [166]. Despite the precise molecular mechanisms through which iron and heme regulate erythropoiesis being poorly understood, one key known regulatory factor in erythroid cells is the heme-regulated eIF2α kinase (HRI). This protein aligns globin mRNA translation with heme availability to ensure balanced hemoglobin synthesis [167]. During heme deficiency, HRI becomes activated and phosphorylated, thereby inhibiting globin mRNA translation and preventing the deleterious precipitation of unbound globins [167]. In regard to enucleation and erythroblast maturation, both are regulated by the transcription factor FOXO3a during which mitochondria aggregate around the nucleus—an event driven primarily by pyruvate and required for enucleation [168]. Notably, RBCs retain mitochondria even after enucleation, allowing heme synthesis and other metabolic activities throughout a cell’s lifespan.

### 3.3. Systemic Heme Recycling, Transport, Sequestration, Degradation, and Elimination

During erythrophagocytosis, HRG1 on phagolysosomal membranes transports heme into the cytosol, where it is catabolized by HO-1 and HO-2 [137]. These enzymes, anchored to the endoplasmic reticulum (ER) membrane with their active sites facing the cytosol, release iron to be stored in ferritin or exported via FPN for new RBC production. HRG1, HO, and FPN are transcriptionally upregulated during erythrophagocytosis [137,169]. In vivo, deletion of HO-1 is embryonically lethal, whereas *Hrg1*-deficient mice survive but accumulate excess heme [170]. Double knockouts of both genes are nonviable, while partial knockout of *Hrg1* in *Hmox1*-null mice causes 40% lethality, indicating an indispensable genetic interaction between both proteins [170]. Recent studies have identified that the cation channel PIEZO1 is an important mediator in macrophage iron homeostasis since mice with a gain-of-function (GOF) of its gene linked to hereditary xerocytosis showed late-onset iron overload [171]. A similar phenotype was observed when the mutation was restricted to macrophages, which was accompanied by elevated RBC turnover, enhanced erythropoiesis, increased ERFE expression, and reduced hepcidin levels [171]. In contrast, expression in hepatocytes of GOF PIEZO1 mutants increased calcium influx and activated ERK signaling, ultimately inhibiting BMP-SMAD signaling and suppressing hepcidin expression [172].

Vascular hemolysis releases free heme and hemoglobin into the bloodstream where they are predominantly cleared by acute-phase proteins [173]. Hemopexin sequesters free heme, forming a complex that is internalized via CD91/lipoprotein receptor–related protein 1 (LRP1) on hepatocytes, macrophages, and neurons, while haptoglobin binds free hemoglobin, enabling uptake through CD163-mediated endocytosis on macrophages [174]. Unlike the haptoglobin–hemoglobin complex, which is ultimately degraded, hemopexin is recycled after delivering heme. Circulating albumin also binds free heme and forms a complex that is endocytosed via the TFR. These heme-scavenging pathways mitigate toxicity and oxidative stress, partly by activating HO-1 [174]. Hemopexin has also been studied extensively in hemolytic disorders such as SCD and together with haptoglobin exhibit neuroprotective effects in ischemia and intracerebral hemorrhage [175]. Once in the liver, heme degradation is mediated by HO-1 and HO-2, the former stress-inducible while the latter is constitutively expressed [176]. Heme oxygenases are primarily expressed in macrophages of the reticuloendothelial system but can be induced in various cells under stress [177]. These enzymes cleave Fe^3+^-PPIX to produce biliverdin, carbon monoxide (CO), iron, and water [139]. Biliverdin is subsequently reduced to bilirubin by biliverdin reductase using NADPH. The iron released is stored in ferritin, while CO participates in signaling and binds iron in hemoproteins. HO-1 transcription is regulated by redox-responsive transcription factors NRF2 and HIFs, and is negatively controlled by BACH1, a heme-binding repressor [178]. Heme oxygenases also play a protective role in various human diseases including cardiovascular, neurodegenerative, neoplastic, metabolic, and inflammatory disorders [179]. In conditions of severe hemolysis, such as SCD, excessive heme is released accompanied by low levels of hemopexin [175].

Alpha-1-microglobulin acts as a secondary scavenger, directing heme to the kidneys, which are key sites for hemoglobin and myoglobin clearance [180]. Hemoglobin interacts with renal proximal tubules through endocytic receptors megalin and cubilin, with megalin mediating reabsorption under normal conditions and cubilin becoming active during hemoglobinuria [181]. However, hemoglobin is nephrotoxic, damaging tubular epithelium, impairing distal tubule function through precipitation, and causing vasoconstriction via nitric oxide scavenging. Injury to proximal tubules, which are rich in mitochondria, exacerbates damage by releasing mitochondrial cytochrome heme [181]. This contributes to acute kidney injury during hemolytic stress and rhabdomyolysis, which are significant causes of mortality in SCD [181].

Hemoparasites like *Plasmodium* crystallize heme into chemically stable hemozoin involving histidine-rich proteins in acidic condition to mitigate toxicity in the absence of a heme degradation system [182], as well as lipids and parasite-derived protein PV5 [183]. Although hemozoin’s structure is well-characterized, its in vivo formation mechanisms remain unclear [183]. In *Hrg1*-deficient mice, hemozoin accumulates in enlarged lysosomes of macrophages that promote heme tolerance. Whether hemozoin serves as a bioavailable iron source during deficiency remains to be elucidated, but targeting its formation offers new therapeutic options for hemolytic anemias [170]. Additionally, lysosomal abnormalities in *Hrg1*-deficient mice suggest possible links between hemozoin formation and lysosomal storage disorders [170].

FLVCR1, identified as a plasma membrane heme exporter [184], has been confirmed in rat renal epithelial cells and erythroid cell lines [185]. *FLVCR1*-deficient mice exhibit defective erythropoiesis, mid-gestation lethality, craniofacial and limb deformities, impaired sensory neuron maintenance, and defective T cell development [186]. FLVCR1 mediates heme-iron recycling by exporting heme from macrophages phagocytosing senescent RBCs. An isoform, FLVCR1b, localized to mitochondria, regulates erythropoiesis by exporting mitochondrial heme and its loss leads to mitochondrial heme accumulation and disrupted erythroid differentiation [187]. FLVCR1a and FLVCR1b also interact with hemopexin to facilitate heme export but mechanisms remain to be elucidated [188]. FLVCR2 (MFSD7C), initially proposed as a cell surface heme importer, is linked to Fowler syndrome, a condition involving proliferative vasculopathy in the brain [189]. Evidence supporting its role in heme transport includes its binding to hemin-conjugated agarose and reduced heme import following *FLVCR2* silencing. Cells expressing FLVCR2 exhibit enhanced heme uptake and increased sensitivity to heme toxicity; FLVCR2 has been localized to mitochondria with a potential role in thermogenesis in response to heme [190]. Notably, FLVCR2 expression in yeast does not rescue growth in heme-deficient strains, highlighting the need for research to clarify its full function in heme transport [191].

MRP5/ABCC5 has been identified as a heme exporter in *C. elegans*, localizing to the basolateral membranes of intestinal cells [192]. As a member of the ABC transporter superfamily, its deficiency results in embryonic lethality and is associated with multidrug resistance, including roles in cancer therapy. Knockdown of *mrp5* in zebrafish leads to severe anemia, highlighting its importance in heme transport. MRP5 is found on the plasma membrane, Golgi complex, and recycling endosomes, thus supporting its involvement in heme export and delivery to hemoproteins [192]. In mammals, its homolog, MRP9, plays a compensatory role in maintaining heme homeostasis [193].

ABCG2, also known as the breast cancer resistance protein, has been suggested as a cell surface heme exporter [194]. *Abcg2* deficiency causes extreme photosensitivity due to accumulation of pheophorbide, a compound structurally similar to PPIX [147]. While ABCG2 exhibits broad substrate specificity for drugs and xenobiotics, its precise physiological substrate has not been reported [195]. However, structural studies point to having a role in transporting heme, PPIX, and other porphyrins [195]. Proteomic analysis has identified eight putative ABC transporters on RBC membranes involved in porphyrin efflux, including mitochondrial transporters ABCB6, ABCB7, ABCB8, and ABCB10 [196]. Of note, ABCB6 initially linked to coproporphyrinogen III transport in the outer mitochondrial membrane has since been found in plasma membranes, endolysosomal compartments, and exosomes of reticulocytes [197]. ABCB6 mediates porphyrin transport, although its substrate is still debated [198]. On the other hand, ABCB7 restores iron homeostasis and cytochrome levels, and is implicated in heme biosynthesis through interactions with FECH, and *ABCB7* mutations are associated with X-linked sideroblastic anemia and mitochondrial iron overload [199]. Similarly, ABCB10 interacts with FECH and the mitochondrial iron importer mitoferrin, further coupling ABC transporters to heme metabolism and mitochondrial function [188].

## 4. Central Nervous System (CNS)

Iron is indispensable for CNS function, contributing to essential processes such as energy metabolism, axonal myelination, and neurotransmitter synthesis. However, its redox activity poses a dual threat, as uncontrolled iron catalyzes production of toxic free radicals that can lead to oxidative stress and neuronal damage. As a result, a tightly regulated system governs iron homeostasis within the CNS to ensure simultaneous availability and detoxification when in excess. Dysregulation of this system is a central factor in several neurodegenerative diseases. The molecular signaling pathways involved in iron regulation and their dysfunction in pathological conditions provide insights into mechanisms of disease progression and opportunities for therapeutic intervention.

### 4.1. Normal CNS Mechanisms of Iron Traffic and Homeostasis

**Figure 4:**
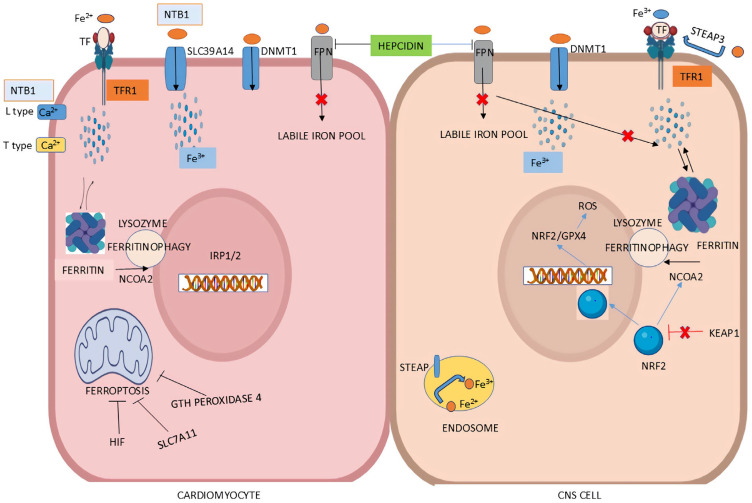
Iron regulation at the cellular level in cardiomyocytes and CNS. (**Left Panel**): In cardiomyocytes, iron binds to transferrin receptor 1 (TFR1). Non-transferrin-bound iron (NTBI) uptake in the heart is primarily facilitated by the ZIP metal ion transporter SLC39A14. Additional NTBI transporters in cardiac cells include L-type and T-type calcium (Ca^2+^) channels. Hepcidin regulates iron levels by promoting ferroportin (FPN) degradation. Ferroptosis is suppressed by hypoxia-inducible factors (HIFs), SLC7A11, and glutathione peroxidase 4 (GPX4)—through enhanced iron uptake and upregulation of TFR1 expression. (**Right Panel**): Iron uptake in CNS occurs via two main pathways: Fe^2+^ is imported by divalent metal transporter 1 (DMT1) after extracellular Fe^3+^ is reduced by ferrireductase STEAP3, while transferrin-bound Fe^3+^ is taken up through TFR-mediated endocytosis. Intracellularly, Fe^3+^ is converted to Fe^2+^ by STEAP3, and heme-bound iron is released by heme oxygenases (HO-1 and HO-2), contributing to the labile iron pool. Excess Fe^2+^, however, generate reactive oxygen species (ROS), leading to oxidative stress. To prevent this, cells store Fe^3+^ in ferritin, which is mobilized during high energy demand via NCOA4-mediated ferritinophagy. Lastly, iron is exported extracellularly as Fe^2+^ through the iron exporter ferroportin. (Illustration by the authors).

Iron enters the CNS primarily through the blood-brain barrier (BBB) in either TF-bound or non-TF-bound forms [200]. The TF/TFR1 complex facilitates Fe^3+^ uptake, where Fe^3+^ is reduced to Fe^2+^ in endosomes by STEAP3 followed by Fe^2+^ transport to the cytoplasm via DMT1 (Figure 4) [201,202]. Iron is stored intracellularly in ferritin cages, composed of heavy (FTH) and light (FTL) chain subunits, where it is kept in a redox-inactive ferric state to prevent oxidative damage from ROS [203,204]. Export of iron is mediated by FPN with the oxidizing assistance of ferroxidases ceruloplasmin and hephaestin. Hepcidin—also modulator of CNS iron homeostasis—binds to FPN and induces its ubiquitin-mediated degradation, preventing further iron release [66,205]. This multi-level regulation ensures iron availability for metabolic needs while mitigating toxicity.

### 4.2. The NRF2/GPX4 Axis in Antioxidant Defense

This axis is pivotal in protecting neuronal cells from ferroptosis. NRF2 is sequestered in the cytoplasm under normal conditions by Keap1. During oxidative stress, NRF2 dissociates from Keap1 and enters the nucleus to induce expression of genes such as *GPX4*, which neutralizes lipid peroxides by reducing lipid hydroperoxides to non-toxic alcohols [204,206]. GPX4’s activity relies on glutathione (GTH), whose levels and biosynthesis are also under NRF2 regulation (Figure 4) [65]. Disruption of the NRF2/GPX4 pathway is linked to disorders such as Parkinson’s and Alzheimer’s disease; and reduction in NRF2 activity severely restricts antioxidant defenses, exacerbating neuronal vulnerability to lipid peroxidation and ferroptosis [64,204]. Elevated ROS through mechanisms like the Fenton reaction further drives the cascade of oxidative damage [202].

### 4.3. BMP/SMAD-Mediated Hepcidin Regulation

BMP6 secreted by liver sinusoidal endothelial cells binds to receptors on hepatocytes to induce phosphorylation of SMAD 1/5/8 proteins, through formation of complexes with SMAD4 that translocate to the nucleus to upregulate hepcidin expression [204,207]. Locally, astrocytes and microglia express hepcidin in response to inflammatory cytokines such as IL-6 that activates the JAK/STAT3 signaling cascade to drive hepcidin transcription during infection or inflammation [66]. High hepcidin levels in the CNS block FPN activity, thus retaining iron within resident cells to limit free iron availability during infection [201]. On the contrary, chronic hepcidin upregulation fosters intracellular iron accumulation that exacerbates iron-dependent oxidative damage and lipid peroxidation, contributing to neurodegeneration in multiple sclerosis and Alzheimer’s [201,205].

### 4.4. Ferritinophagy and Iron Availability

Selective autophagic degradation of ferritin, termed ferritinophagy, provides labile iron needed for cellular functions. NCOA4 mediates ferritin trafficking to autophagosomes for degradation, releasing stored iron into the cytoplasm (Figure 4) [202,208]. Dysregulation of NCOA4 expression or activity disrupts iron homeostasis and the resulting excessive ferritinophagy enhances cytoplasmic iron levels, promoting ROS, higher lipid peroxidation and ferroptosis [203]. Conversely, insufficient ferritinophagy in conditions of iron excess impair the cell’s ability to utilize stored iron effectively exacerbating neurotoxicity. NRF2 also influences ferritinophagy by regulating NCOA4 and ferritin heavy chain expression, providing a control mechanism over intracellular iron pools [203]. This reduction of labile iron through reduced ferritin degradation has shown protective effects against ROS-mediated damage in experimental disease models [65,200].

### 4.5. Neuroinflammation and Iron Dysregulation

Neuroinflammation provides another layer of CNS iron regulation. Activation of microglia during inflammatory states increases hepcidin secretion and promotes iron sequestration within cells [203]. This localized retention contributes to a feed-forward mechanism of oxidative stress and neurotoxicity, particularly in multiple sclerosis and traumatic brain injury. Chronic inflammation linked to SCD, accelerates these processes by enhancing hepcidin expression and perpetuating iron imbalance [208,209].

### 4.6. Role of Iron in CNS Aging and Neurodegenerative Diseases

Iron metabolism in the CNS is tightly regulated to support vital processes like myelination, mitochondrial function, and neurotransmitter synthesis while avoiding oxidative stress. With age, iron accumulates in certain brain regions, potentially amplifying inflammation and contributing to neurodegeneration [67]. Reports indicate that iron dysregulation is a hallmark of several diseases, including both acquired conditions like Parkinson’s and Alzheimer’s, and congenital disorders such as pantothenate kinase-associated neurodegeneration and Friedreich’s ataxia [67,210]. For instance, excessive iron in the substantia nigra of Parkinson’s patients promotes α-synuclein aggregation and neuronal loss due to oxidative damage [211,212]. Similarly, Alzheimer’s pathology is associated with tightly bound iron in cortical areas where oxidative stress and ferroptosis mediate amyloid-beta toxicity and neuronal death [211]. This highlights the critical need for balanced iron homeostasis during brain aging and pathology.

### 4.7. Mechanisms Underlying Iron-Induced Neurotoxicity

Iron’s capacity to transition between Fe^3+^ and Fe^2+^ oxidation states underlies its dual role as both physiologic requirement and a threat to cells. Dysregulated iron amplifies production of ROS, which regardless of organ system oxidizes lipids, damages DNA, and disrupts protein functions [213]. This oxidative stress injures neurons and exacerbates neuroinflammatory pathways. For example, the brain’s choroid plexus, which regulates iron exchange at the blood-cerebrospinal fluid barrier, has been implicated in conditions of iron overload where iron deposition contributes to localized neurodegeneration [67].

### 4.8. Iron Accumulation and Possible Therapies

Accumulated iron in the CNS does not merely reflect pathology but may also actively propagate it. In Alzheimer’s, iron is sequestered into non-bioavailable forms, impairing mitochondrial processes and neuronal metabolism [211]. Downregulation of FPN exacerbates iron retention, driving oxidative stress and damage. Likewise, disruptions in genes responsible for iron transport and mitochondrial function, such as *PITRM1* in Alzheimer’s, point to an interplay between iron metabolism and neurodegeneration [211].

Advances in understanding the molecular pathways of iron dysregulation in the CNS have created avenues for targeted therapies. Enhancing NRF2 activity pharmacologically or modulating BMP/SMAD-induced hepcidin expression can recalibrate iron homeostasis and mitigate oxidative stress. Iron chelators such as deferoxamine, can reduce iron overload and prevent lipid peroxidation [66,204]. Ferritinophagy inhibitors targeting NCOA4 or strategies to restore mitochondrial iron balance further broaden the scope for therapeutic interventions. Furthermore, enhancing the antioxidant defenses of NRF2/GPX4 pathways mitigate ferroptosis and reduce oxidative stress [213]. In this way, compounds that modulate ferritinophagy or regulate iron transport across the BBB alleviate the toxicity of iron overload [67,211]. Additionally, the relationship between trace metal toxicity (e.g., iron and manganese) and their combined impact on neural integrity provide insights into developing new therapies [213]. These clinical frameworks hold promise for addressing iron-driven neurotoxicity in neurodegenerative diseases.

## 5. Iron Metabolism in the Cardiovascular System

### 5.1. Iron Uptake

Iron binds to TFR1 and is endocytosed by clathrin in cardiomyocytes [214,215]. Decreases in iron levels in cardiac cells increases expression of IRP1 and IRP2 [215]. Mice lacking cardiac TFR1 develop severe and lethal cardiomyopathy [216]. In hemochromatosis, cardiac iron overload is a late manifestation. Of note, the heart’s NTBI uptake is 10–100 times lower compared to other organs and it is mediated by SLC39A14, a ZIP metal ion transporter [217]. However, the main function of ZIP transporters is zinc transport [217,218]. The other NTBI transporters in heart are mainly L-type and T-type Ca^2+^ channels (Figure 4) [120]. In iron overload, L-type Ca^2+^ channels play the main role in uptake of Fe^2+^ into cardiomyocytes and possibly drive initiation of pathologic changes [219].

### 5.2. Hepcidin–FPN Axis and Iron Export Regulation in Cardiovascular System

Hepcidin exerts its action by degrading FPN in macrophages, intestine and liver as discussed earlier [220]. In cardiac ischemic and reperfusion injuries both cellular and mitochondrial functions are affected, leading to higher iron stores in mitochondria followed by oxidative damage of cardiomyocytes [221]. Thus, there is a direct association between heart failure and cellular and mitochondrial injuries. Nevertheless, the degree of increased mitochondrial iron stores depends on cardiomyopathy type, specifically if it is ischemic/reperfusion, secondary to hemochromatosis, or chemotherapy-induced [221]. Both iron deficiency and overload states are involved in development of heart failure, and dysfunctional mitochondria in these settings produce ROS that worsens pathology [221].

### 5.3. BMP/SMAD Pathway in Hepcidin Regulation

BMPs mediate a critical role in regulating organogenesis and cardiovascular function [222]. In the cardiovascular system, activated macrophages potentiate atherosclerosis by expressing BMP2, BMP4, and BMP6, which are pro-atherogenic, whereas BMP9 is anti-atherogenic [222]. The BMP6 inhibitor, LDN-193189, suppresses hepcidin and increases FPN expression in macrophages leading to decreased iron storage, thus functioning as anti-atherogenic agent [223]. LDN-193189 prevents atherosclerosis by inducing expression of ABC transporters resulting in lower intracellular iron and oxidative stress [224]. This favors use of this type of agents in clinical trials looking at atherosclerotic patients.

### 5.4. Ferroptosis in Cardiomyocytes

The involvement of ferroptosis in cardiovascular disease is well established [225]. Stress-induced lipid peroxidation promotes ferroptosis which mediates cardiomyocyte injury during myocardial infarction, ischemic/reperfusion conditions, and heart failure [226]. Ferroptosis can be prevented by HIF through increases in iron uptake and expression of TFR1 (Figure 4) [227]. FTH and FTL subunits expression vary among organs, for example FTL-rich ferritin is seen in liver and spleen, whereas FTH is found in heart [228]. FTH has ferroxidase activity and mice lacking FTH1 in cardiomyocytes, when exposed to a high-iron diet, experience increased lipid peroxidation and hypertrophic cardiomyopathy [229]. Also, FTH-deficient cardiomyocytes have reduced expression of the ferroptosis regulator SLC7A11, a subunit of the heterodimeric cystine-glutamate antiporter, that when overexpressed is associated with increased synthesis of GTH and inhibited lipid peroxidation, thus preventing ferroptosis [228,230]. This causes injury to cardiomyocytes reducing SLC7A11 and GTH-peroxidase 4 levels, and doing so suppressing ferroptosis [231]. In this regard, ferroptosis inhibitors ferrostatin-1 and liproxstatin prevent ferroptosis in liver, kidney, brain, and heart in mice [226]. Ferroptosis also occurs in sepsis-induced cardiac inflammation. Treatment with ferrostatin-1 improves cardiac dysfunction by inhibiting the TLR4/ nuclear factor kappa B (NF-κB) signaling pathway preventing cardiac injury in sepsis [232].

### 5.5. Iron Deficiency and Heart Failure

The biomarkers fatty acid binding protein-4, growth differentiation factor-15, NT-proBNP, osteopontin, ST2 protein, tumor necrosis factor receptor-1, and TFR1 are increased in iron deficiency states, whereas paraoxonase-3 and tartrate-resistant acid phosphatase type-5 are downregulated [233]. Consequently, measuring these markers can confirm iron deficiency and identify possible underlying cause(s) [233]. In this way, a ten-year-long study from the National Health and Nutrition Examination Survey showed that either intravenous or dietary iron intake benefits adult patients with heart failure [234].

## 6. Iron Metabolism in Lungs

### 6.1. Iron Metabolism and TBI Uptake

The lung has both extracellular and intracellular iron distribution mechanisms [235]. To emphasize the importance of iron in the lung, both TF and TFR are expressed in bronchial epithelium, type II alveolar cells, macrophages, and bronchus-associated lymphoid tissue [236]. Alveolar epithelial cells and macrophages also express DMT1, a transporter of nonheme iron and endosomal iron from the TF-TFR1 complex into the cytoplasm regulated by the IRP/IRE system [237]. Finally, pulmonary epithelial cells also have antioxidant molecules that prevent oxidative stress due to iron among a number of elements/molecules protecting the tissue from inflammation and/or injury [235].

### 6.2. NTBI in Lungs

The key NTBI importers are DMT1, ZIP14, ZIP8, and lactoferrin receptors [238]. DMT1 and DCYTB are expressed by pulmonary epithelial cells; while ZIP14, during iron overload states, mediates NTBI uptake by hepatocytes, heart, pancreas, and pulmonary epithelial cells [238]. ZIP8 (or SLC39A8) is a divalent metal ion importer expressed in lung, induced under inflammatory conditions that if absent leads to elevated splenic iron levels and hypoferremia; interestingly, *ZIP8^−/−^* mice do not develop acute lung injury (ALI) during markedly reduced serum iron, suggesting that this receptor is involved in iron recycling [239]. Natural resistance-associated macrophage protein-1 (or SLC11A1) is involved in NTBI uptake in pulmonary macrophages and neutrophils by sequestering excess iron in the immune system and transporting it as needed [240]. LRP1 is an iron bound glycoprotein, belonging to the TF family that functions as iron chelator [238,241]. This receptor actively removes iron, thus decreasing its pro-inflammatory effects [242], such as in pulmonary alveolar proteinosis when lactoferrin levels exceed TF [243], and during bacterial and fungal infections when iron chelation is therapeutic [244].

### 6.3. Hepcidin–FPN Axis (Iron Export Regulation) in Lungs

Hepcidin is expressed in lower concentration in the lungs compared to other organs [245]. A hepcidin-knockout model has shown that its absence led to increased FPN and FTL1 mRNA expression in epithelial cells, resulting in higher alveolar iron export and elevated iron in macrophages [246]. *HAMP* expression by alveolar macrophages can be induced by lipopolysaccharide stimulation through upregulation of DMT1 and downregulation of FPN, suggesting that there is decreased iron efflux in inflammatory states [247]. Indeed, *HAMP* expression increases in alveolar macrophages during inflammation and degradation of FPN decreases iron efflux, meaning that hepcidin protects the lungs against infection or inflammation by creating an iron-limited environment and decreased iron-mediated oxidative lung injury [247,248]. Even though iron has no direct effect on *HAMP* expression, it upregulates DMT1 and FPN1. Therefore, iron loading of the lungs is regulated by a hepcidin–FPN independent mechanism [247,249].

### 6.4. Ferroptosis in the Lungs

Inhaled oxidants or injured pulmonary cells lead to ROS formation. Pulmonary diseases such as acute respiratory distress syndrome are pro-oxidant states with increased body iron stores [238]. Among interleukins, IL-4 and IL-13 are key inflammatory intermediates causing robust lung inflammation in response to insults such as iron [250]. SLC7A11 is also downregulated by p53 causing decreases in GTH synthesis, further exacerbating ROS and ferroptosis [250]. Interestingly, oleic acid-induced ALI in animals is characterized by iron overload, decreased GTH, and accumulation of malondialdehyde indicating that ferroptosis is part of this type of lung injury [251]. One of the significant changes in the lungs is that iron excess induces activation of fibroblasts, causing pulmonary fibrosis through induction of the TGF-β-TAZ-TEAD signaling pathway resulting in fibroblast-to-myofibroblast conversion [252]. Radiation-induced lung injury also causes ROS accumulation, promoting ferroptosis in epithelial cells by increased expression of SLC7A11, GPX4 downregulation, increased in malondialdehyde and total iron when exposed to lipopolysaccharide and ferrostatin-1 [253]. Similarly, uridine phosphorylase 1 works as ferroptosis inhibitor via the NRF2 signaling pathway [254]. Blood transfusions promote ferroptosis by driving blood transfusion-associated ALI; thus, iron chelators and anti-inflammatory drugs are helpful in this setting [250]. These pathways are summarized in Table 1.

## 7. Iron Metabolism in Reproductive System

Spermatogenesis is an iron-dependent process [255]. In particular Sertoli cells have high iron requirements due to their enhanced proliferative baseline phenotype [256]. Developing spermatocytes, however, are highly vulnerable to oxidative stress requiring tight regulation of iron metabolism. Gonads are indirectly responsive to iron overload because in diseases such as hereditary hemochromatosis and hemoglobinopathies, increased iron is deposited in pituitary gonadotrophs, decreasing release of gonadotrophins and resulting in hypogonadotropic hypogonadism [257]. Over time this results in infertility and delayed puberty.

### 7.1. Transferrin and Non-Transferring Bound Iron

Extracellular or exogenous iron uptake is mainly mediated by the TF-TFR1 pathway in Sertoli cells [258]. Testicular transferrin is synthesized by seminiferous epithelium resulting in iron flow from the basal layer to the luminal spermatogonia [259]. Seminiferous epithelial cells express TFR1 which readily takes in iron from circulation through the blood–testicular barrier [259]. Additionally, ceruloplasmin is also involved in this process and in oxidizing Fe^2+^ [260]. DMT1 expressed mainly in the intracellular compartment of seminiferous tubules’ Sertoli cells mediates most of the iron handling [261]. However, mature germ cells also express DMT1, but this expression is dependent upon the spermatogenesis stage since iron requirement varies from one stage to the next [262].

### 7.2. Hepcidin–FPN Axis (Iron Export Regulation) in Reproductive System

Iron is stored as ferritin within cells and utilized during spermatogenesis. It is found in two sites in the testis: mitochondrial (in interstitial Leydig cells and germinal cells in seminiferous tubules) and cytosolic (interstitium) [261]. Sertoli cells actively phagocytose spermatozoa failing to reach their final differentiation stage [261]. Iron contained in phagocytosed spermatogonia is recycled by Sertoli cells so it can be used for next cycle of spermatogenesis. FPN present on the basolateral surface of seminiferous tubules exports excess iron from the luminal surface back to circulation [262]. As an added safety mechanism, both blood–testicular barrier and FPN prevent iron overload in the testis [263].

### 7.3. Ferroptosis in Testis

Ferroptosis is important during spermatogenesis. Mice lacking GTH-synthetase, final step in the synthesis of GTH, results in males with markedly reduced fertility [264]. With increasing age, this enzyme deficiency gets progressively worse so that lower GTH leads to ROS accumulation and lipid peroxidation, thus promoting ferroptosis. Therefore, ferroptosis not only disrupts meiosis, but leads to disordered acrosomal development [264].

## 8. Endocrine

### 8.1. Mechanisms of Iron Toxicity

Excess iron in endocrine tissues equally promotes oxidative stress and inflammation through ROS formation. This oxidative stress damages cells in the pancreas, pituitary, and thyroid, among other glands. ROS damages the ER, leading to ER stress and activation of the unfolded protein response in endocrine glands [265]. Interestingly, NTBI often enters endocrine cells using calcium channels, where it causes cytotoxicity via ROS. These oxidants mediate damage by stimulating FOXO3a, which upregulates ferritin, altering the LIP and disrupting hormonal synthesis [266]. Activation of NADPH-oxidase generates superoxide radicals that amplify ongoing inflammation and cellular injury [267]. TRAF6 ubiquitin-ligase and Akt pathway inhibition by ROS promote apoptosis of endocrine cells, including insulin-secreting pancreatic cells [266]. Under normal conditions, iron-dependent prolyl hydroxylases mark HIF-1α for degradation. However, iron excess inhibits these hydroxylases, stabilizing HIF-1α and promoting its accumulation [268]. This stabilization leads to increased expression of genes involved in angiogenesis, glycolysis, and cell survival, potentially contributing to tumor progression and metastasis in thalassemia patients [268]. As a result, therapeutic interventions focusing on iron chelation, and novel approaches like hepcidin mimetics and anti-sense oligonucleotides such as TMPRSS6 can modulate hepcidin levels mitigating iron toxicity and reversing endocrine insults [269]. Melatonin has also been reported to mitigate oxidative damage via its antioxidant properties, though its effects appear iron-independent [270]. Moreover, iron chelators beyond reducing intracellular iron, improve endocrine function in transfusion-dependent patients [271]. However, chronic elevation of hepcidin due to inflammatory stimuli exacerbate endocrine iron dysregulation further, causing or worsening diabetes and hypothyroidism. Therefore, strategies combining iron chelation with hormone replacement address both systemic and endocrine sequelae of iron overload [272].

### 8.2. Pituitary and Hypothalamus

Iron accumulation in the anterior pituitary gland reduces gonadotropic hormone production, causing hypogonadism and delayed puberty in patients with transfusion-dependent thalassemia or hereditary hemochromatosis [273,274]. Pituitary dysfunction is particularly common in transfusion-dependent β-thalassemia patients [275]. Evidence has shown that transfusion-dependent patients experience hypogonadotropic hypogonadism, mainly due to ferritin accumulation in the anterior pituitary, suppressing gonadotropin-level pathways especially those producing growth hormone (GH) and thyroid-stimulating hormone (TSH) [276]. Hypogonadotropic hypogonadism, driven by pituitary hemosiderosis is a direct result of iron-related impairment of gonadotropin secretion [277,278]. Anatomically, the pituitary gland has greater susceptibility to iron overload due to its intense vascularization and high metabolic activity. This is compounded by its dense expression of TFR making it particularly iron-sensitive [279]. Additionally, iron-induced downregulation of the hypothalamus–pituitary–gonadal axis occurs through iron deposition in gonadotrophs. This causes suppression of gonadotropic releasing hormone from the hypothalamus [280]. Furthermore, cells essential for the synthesis of luteinizing hormone and follicle-stimulating hormone, experience impaired secretion secondary to ROS-related mitochondrial dysfunction [276]. Finally, iron overload hastens cellular aging processes by shortening telomeres, further exacerbating tissue dysfunction. Not unexpectedly, impaired endocrine responses also linked to iron toxicity include thyroid dysfunction, hypoparathyroidism, and adrenal insufficiency [278].

Bone deformities and growth retardation observed in iron-overloaded patients are linked to iron’s interference with GH–insulin-like growth factor-1 (IGF-1) axis [281]. Excess iron deposition in the liver and impairment of IGF-1 production together diminish the anabolic effects of GH, delaying skeletal growth and maturation [281]. Iron also disrupts osteoblast differentiation and activity through ROS-mediated inhibition of Wnt/β-catenin signaling, contributing to significant osteoporosis [281]. This iron-induced osteoporosis occurs through upregulation of osteoclast activity and suppression of osteoblast function; this involves the RANKL/osteoprotegerin pathway, as observed in SCD and thalassemia patients [282].

### 8.3. Thyroid and Parathyroids

In the thyroid, iron excess reduces production of TSH, free thyroxine (T4), and inhibits IGF-1 production by the liver, contributing to growth abnormalities [283,284]. Thyroid cells are particularly sensitive to ROS, as hydrogen peroxide, critical in thyroid hormone biosynthesis, amplifies oxidative injury during iron overload conditions [270], that can similarly affect parathyroid glands [275]. High oxidative load also impairs iodide transport, an essential step for synthesis of T4 and triiodothyronine [285]. This dysfunction correlates with altered levels of TSH and free T4 seen in patients with chronic iron overload [286]. Moreover, this dysregulation is observed in diseases like juvenile hemochromatosis, which presents with early-onset hypothyroidism due to severe glandular iron deposition [287]. Central hypothyroidism is similarly associated with impaired TSH production due to iron-induced pituitary damage, as seen in children with transfusion-dependent anemia or SCD [288]. Notably, primary thyroid dysfunction from iron-induced oxidative stress and glandular inflammation commonly manifests as subclinical hypothyroidism, marked by elevated TSH and normal T4 levels [285,286]. Impaired fibroblast growth factor-23 secretion is also seen in hypoparathyroid thalassemic patients, suggesting that iron overload disrupts both hormonal and mineral regulatory pathways in bone-parathyroid interactions [289]. As a result, this disruption worsens the clinical spectrum of hypocalcemia and hyperphosphatemia, characteristic of iron-overloaded states [289].

### 8.4. Pancreas

The loss of β-cell function is a critical factor in the development of diabetes in patients with hemoglobinopathies. Excessive iron deposition impairs β-cell function, leading to glucose dysregulation, insulin resistance, and overt diabetes mellitus, often termed “bronze diabetes” [290]. The resulting pancreatic siderosis is an early indicator of endocrine imbalance, aligned with hyperglycemia and dysregulated glucose metabolism [291]. The oxidative stress induced by iron overload interferes with insulin signaling pathways through modifications of insulin receptor substrates, which impairs their ability to propagate insulin signals [265]. Additionally, ROS activates stress kinases such as c-Jun N-terminal kinase (JNK), and p38 mitogen-activated protein kinase (MAPK), further inhibiting insulin signaling by phosphorylating serine residues [265]. Chronic ER stress also triggers apoptotic pathways, reducing β-cell mass and insulin secretion [292]. Furthermore, activation of signaling pathways such as protein kinase R-like ER-kinase and inositol-requiring enzyme-1 further disrupts β-cell function [293]. NTBI transporter ZIP14 facilitates parenchymal iron accumulation in pancreatic acinar cells [294]. NTBI, particularly its LIP fraction, penetrates pancreatic cells through unregulated calcium channels, amplifying intracellular iron overload and associated oxidative damage [295,296]. Of note, chronic inflammatory responses further compound this damage. Excess iron activates inflammatory pathways, as shown by increased IL-1β, IL-6, inducible nitric oxide synthase expression, as well as neutrophil infiltration [297]. Chronic iron overload continuously stimulates release of pro-inflammatory cytokines IL-6 and TNF-α, promoting tissue fibrosis and triggering β-cell apoptosis [298]. Chronic inflammation during iron overload also promotes development of acinar-to-ductal metaplasia in pancreas, which are precursors to neoplastic transformations linked to increased ROS and altered p53 activity [299]. These factors equally activate pancreatic stellate cells, further driving fibrosis and acinar atrophy, leading to progressive loss of exocrine function as well [297]. Additionally, ongoing iron deposition in pancreatic islets potentiate β-cell impairment [297]. Pancreatic iron deposition is also associated with increased hepatic insulin resistance due to decreased clearance of insulin leading to systemic hyperinsulinemia and metabolic dysfunction [298]. In response to iron overload, phenolic compounds such as ferulic acid and its derivatives enhance nuclear translocation of NRF2, upregulating antioxidant defense mechanisms within the pancreas including genes such as *HO-1* and *GPX4*, which are protective against ROS-induced damage [300]. Unfortunately, *HFE* mutations, such as *C282Y* and *H63D*, disrupt hepcidin regulation, facilitating increased intestinal absorption of iron and diffuse deposition in organs, especially pancreas [301].

### 8.5. Gonads and Adrenals

ROS activates the p53 pathway causing DNA damage and p53-mediated apoptosis of endocrine cells, particularly in pituitary and testicular tissue [302]. Oxidative stress profoundly affects ovarian follicles and steroidogenesis through disrupted aromatase activity and impaired estradiol synthesis [303]. Male gonads experience Leydig cell dysfunction disrupting testosterone biosynthesis and luteinizing hormone signaling [276]. At the molecular level, iron-regulated signaling pathways include iron-mediated activation of stress-responsive pathways such as JNK, p38MAPK, and NF-κB [281]. Once activated, these pathways trigger apoptotic signaling and cytokine production, expanding endocrine dysfunction and inflammation. Connections between iron overload and adrenal insufficiency are highlighted in patients with β-thalassemia major, where adrenal insufficiency has been detected in up to half of patients, due to iron-mediated mitochondrial damage impairing cortisol biosynthesis pathways [304]. Adrenal dysfunction in SCD is similarly attributed to microvascular obstruction and repeated vaso-occlusive episodes, which result in ischemic injury to small blood vessels of adrenal glands [304]. These events, compounded by iron deposition from repeated blood transfusions, profoundly disrupt adrenal steroidogenic machinery [305]. Finally, ROS-mediated disruption of the steroidogenic acute regulatory protein impairs cholesterol transport within mitochondria, exacerbating hypocortisolism even further [293].

## 9. Therapeutic Approaches to Improve Iron Excess

It is clear from the data presented that approaches that reduce iron deposition are needed to prevent multi-systemic complications associated with iron excess. Iron chelators have been used as first-line agents to reduce iron stores in patients such as those with SCD. The iron chelator deferasirox has been shown to effectively reduce ferritin concentration and overall oxidative stress, including telomere length, in dialysis-dependent patients [306]. In patients with thalassemia major, exposed to regular simple transfusions iron chelators, deferoxamine and deferiprone have shown to be similarly effective to deferasirox in reducing ferritin and overall iron stores, leading to net iron excretion with few adverse events [307]. However, a prevailing challenge to chelation is patient adherence due to adverse events associated with these agents. Notably, a meta-analysis of available clinical trials has indicated that adherence to chelation is relatively high, and a combination of agents such as deferiprone and deferasirox results in greater adherence to chelation with few adverse events in patients with hemoglobinopathies [308]. Importantly, combination therapy with two chelators such as deferiprone/deferasirox and deferiprone/deferoxamine has been reported to be effective [309].

A different approach has sought to use vitamin E’s antioxidant capabilities to address oxidative stress in iron overload. For instance, a randomized trial (NCT06509581) showed that vitamin E potentiated deferasirox’s iron reduction effect and ROS decreases as indicated by decreased glutathione, superoxide dismutase, glutathione peroxidase, and peroxiredoxin-2 [310]. In transfusion-dependent MDS patients the chelator deferiprone decreased iron-induced oxidative markers including LIP, without signs of neutropenia or agranulocytosis [311]. In children, even though use of chelation can be challenging, the CALYPSO randomized trial (NCT02435212) showed that deferasirox was well-tolerated with adverse events limited to pyrexia, higher urine protein/creatinine ratio, and cases of upper respiratory infections [312]. Likewise, results from the FIRST trial looking at iron overloaded sickle cell pediatric patients indicated that deferiprone and deferoxamine decreased liver iron >3 mg/g of dry weight and serum ferritin concentration with adverse events mostly limited to higher liver transaminases, vomiting, and pyrexia [313].

For patients with iron overload due to hepcidin deficiency, use of peptide mimetics of this protein represents a therapeutic option to improve their iron absorption baseline. Rusfertide is such a mimetic that has been shown to significantly decrease not only iron stores but the number of phlebotomies needed by hemochromatosis patients over the length of treatment duration (NCT04202965) [314]. Rusfertide has also been shown to reduce overall iron by decreasing erythrocytosis in patients with polycythemia vera (PV) (NCT04057040) [315]. Furthermore, an aqueous formulation of rusfertide showed dose-dependent reduction in serum iron and transferrin-iron saturation in PV patients [316]. Its safety was confirmed with a report of minimal adverse events using a subcutaneous injection of rusfertide into healthy subjects [316].

Another therapeutic target is FPN. In a SCD mouse model, the FPN inhibitor vamifeport reduced hemoglobin concentration, intravascular hemolysis and inflammation thus preventing disease crises [317]. This agent works by acting directly on the FPN–hepcidin axis. In a mouse model of hemochromatosis, vamifeport administration resulted in significant sustained liver and serum iron reduction, inducing transient hypoferremia after a single dose [318]. This agent, due to its minimal adverse events and effectiveness in a β-thalassemia mouse model, may hold promise for patients with this type of ineffective erythropoiesis through its reduction of transferrin-iron saturation [319,320]. Importantly, just as indicated above for rusfertide, vamifeport has proven safe in healthy subjects with dose-dependent improvements in iron elimination [321] Finally, a developing area of research is through targeting of protein kinase C which through its hyperactivation leads to iron overload in diabetics [322]. This protein acts a positive regulator, increasing exocytotic FPN trafficking and decreasing endocytic trafficking of FPN at rest, suggesting that its pharmacological inhibition results in iron improvement in hemochromatosis [322].

Without a doubt a new generation of gene therapies holds promise to cure patients with hemoglobinopathies. Products such as lovotibeglogene autotemcel (Lyfgenia) and exagamglogene autotemcel (Casgevy) which have been approved for use by the Food and Drug Administration to treat SCD and transfusion-dependent β-thalassemia (the latter) represent opportunities to free patients from the chains of a life of transfusions and high iron exposure [323]. These gene-editing therapies, use the patient’s own cells which are either transduced with lentiviral vectors that encode for beta-globin gene(s) to generate hemoglobin incapable of sickling (Lyfgenia) or via CRISPR/Cas9 leading to higher hemoglobin F production (Casgevy) are the latest example of personalized medicine. These and other products currently in clinical trials will result in fewer patients having vaso-occlusive crises and complications associated with these chronic anemias.

## 10. Conclusions

Iron’s requirement for maintaining a large number of cellular functional processes is evident. The generation of transporters that establish links between sites of absorption to those sites where iron is recycled and biosynthetically required exemplifies a complex of multilayer regulatory pathways required to control its intake, utilization, export, storage and recycling. Beginning with mediators that bind it, reduce it, transport it across the cytosol to organelles including the mitochondria, to elimination from the cell, is orchestrated through an intricate and at times redundant set of pathways keeping iron concentration fine-tuned for proper physiology to be maintained. These pathways are tissue-specific and even result in gene expression to better control iron disposition. Thus, iron bioavailability is paramount to the organism and its deleterious effects seen when overwhelming these pathways results in profound pathological changes and extensive tissue damage through processes such as ROS generation. This is why despite current knowledge, additional research to get a comprehensive and better understanding of the negative effects secondary to iron overload is needed. Thankfully, iron chelation has shown to be helpful to minimize iron-associated complications and gene-editing therapies will likely result in quality-of-life improvements for patients requiring chronic transfusions due to hemoglobinopathies. Nevertheless, there ought to be a continuous development of targeted therapies to mitigate or prevent the severe complications associated with iron dysregulation.

## Figures and Tables

**Table 1:** Mediators involved in iron metabolism and pathologic mechanisms caused by iron dysregulation.

	Physiological Iron and Pathways Involved	Pathological	RX ^1^
CNS	– TF-bound or non-TF-bound forms – TF/TFR1 complex facilitates Fe^3+^ uptake – Fe^3+^ reduced to Fe^2+^ in endosomes by STEAP3 – Fe^2+^ transported to cytoplasm via DMT1 – Iron stored intracellularly in ferritin cages (FTL/FTH) – Iron export mediated by FPN – Hepcidin binds FPN and induces ubiquitin-mediated degradation.		Iron chelators: deferoxamine, has shown neuroprotective activity reducing iron overload and preventing lipid peroxidation
	NRF2/GPX4 Axis: During oxidative stress, NRF2 dissociates from Keap1 and enters nucleus to induce gene expression such as *GPX4* to neutralize lipid peroxide	Disruption of NRF2/GPX4 associated with Parkinson’s and Alzheimer’s Reduced NRF2 activity weakens antioxidant defenses. Increased neuronal susceptibility to lipid peroxidation and ferroptosis.	
	BMP/SMAD-mediated hepcidin axis regulation: – Astrocytes and microglia express hepcidin in response to inflammatory cytokines like IL-6 – IL-6 activates JAK/STAT3 pathway, inducing hepcidin during infection or inflammation.	High CNS hepcidin inhibit FPN, reducing free iron during infection Chronic hepcidin upregulation → intracellular iron buildup Excess iron accumulation → neurodegeneration in multiple sclerosis and Alzheimer’s	BMP6 inhibitor, LDN-193189, suppresses hepcidin and increases FPN expression
	Ferritinophagy: NCOA4 mediates ferritin trafficking to autophagosomes for degradation, releasing stored iron into cytoplasm	Dysregulation of NCOA4 expression or activity disrupts iron homeostasis → excessive ferritinophagy	
	Mitochondrial iron regulation: – Fe-S cluster biosynthesis and energy generation via oxidative phosphorylation – Aconitase activity	Excessive mitochondrial iron → elevated ROS levels Mitochondrial membrane depolarization → impaired ATP production	
	Iron in aging CNS/neurodegenerative diseases	Iron accumulates in aged brain → amplifies inflammation and neurodegeneration	
Heart	– Iron binds TFR1, endocytosed by clathrin in cardiomyocytes – Decreased iron in cardiomyocytes increases expression of IRP1 and IRP2 – NTBI uptake mediated by SLC39A14 – L-type Ca^2+^ channels main role in Fe^2+^ uptake into cardiomyocytes		
	Hepcidin–FPN axis/iron export regulation	Cardiac ischemic and reperfusion injuries → higher iron stores in mitochondria followed by oxidative damage of cardiomyocytes	
	BMP/SMAD axis in hepcidin regulation: – Activated macrophages promote atherosclerosis via pro-atherogenic BMP2, BMP4, BMP6 – BMP9 anti-atherogenic effects → counters atherosclerosis		BMP6 inhibitor, LDN-193189, → anti-atherogenic agent
	Ferroptosis: – Upregulated SLC7A11 enhances GTH synthesis, boosting antioxidant defense – Increased GTH inhibits lipid peroxidation, prevents ferroptosis	Reduced SLC7A11 and GTH-peroxidase 4 levels suppress ferroptosis → cardiomyocyte injury	
Lungs	– DMT1 transports dietary nonheme iron and endosomal iron (from TF-TFR1 complex) into cytoplasm, regulated by IRPs/IREs – Pulmonary epithelial cells DMT1 and DCYTB import NTBI – ZIP8 (SLC39A8) expressed in lung and upregulated during inflammation	In iron overload → NTBI uptake predominantly by hepatocytes, heart, pancreas, and pulmonary epithelial cells	
	Hepcidin–FPN axis: – Increased FPN and FTL1 expression in lung epithelial cells enhances alveolar iron export – Iron accumulates in alveolar macrophages due to elevated epithelial iron efflux – Inflammation → *HAMP* expression in alveolar macrophages – Hepcidin degrades FPN, reduces iron efflux from macrophages, promotes iron retention	Iron loading of lungs regulated by hepcidin-FPN independent mechanism	
	Ferroptosis	Downregulation of SLC7A11 reduces GTH synthesis, increasing ROS, promoting ferroptosis Radiation-induced lung injury → ROS buildup, further drives ferroptosis Blood transfusions → ↑ ferroptosis, transfusion-related acute lung injury	
Liver	EPO–ERFE–Hepcidin axis: Hypoxia → ↑ EPO (via HIF-2) → ↑ ERFE → ↓ hepcidin → ↑ iron availability → ↑ erythropoiesis	Ineffective erythropoiesis (e.g., β-thalassemia): ↑ ERFE → ↓ hepcidin → iron overload → ROS generation and tissue damage When found with renal disease → delayed ERFE induction by EPO → ineffective iron mobilization → anemia	
	BMP-SMAD1/5/8 signaling: Liver iron → ↑ BMP2/6 → BMPR → SMAD1/5/8 → SMAD4 complex → ↑ *HAMP* expression	In hereditary hemochromatosis: ↓ HFE → impaired BMP/SMAD activation → ↓ hepcidin → iron overload	
	ERFE expression: Erythroblasts in MDS → mutant ERFE with preserved function → ↓ Hepcidin	MDS-RS with SF3B1 mutations: ↑ ERFE → systemic iron overload	
	NRF2 oxidative stress response: ROS → NRF2 release from Keap1 → nucleus translocation → expression of antioxidant and iron-regulating genes (HO-1, ferritin, FPN)	In cancer: Hyperactive NRF2 → protects tumor cells from ROS, chemotherapy, radiotherapy, and ferroptosis	NRF2 inhibition may restore ferroptosis and sensitize tumors to therapy
	IL-6/STAT3 pathway: inflammation → IL-6 → IL-6R/gp130 → JAK/STAT3 → ↑ Hepcidin → Iron sequestration	Chronic inflammation/autoimmunity: Overactive pathway → anemia of chronic disease (ACD)	IL-6R inhibitors in rheumatoid arthritis, cancer
	ZIP14-mediated NTBI uptake: ZIP14 imports NTBI → protects other tissues	Prolonged ZIP14 activity → hepatic iron overload → fibrosis, cirrhosis, hepatocellular carcinoma Regulated by IL-6: inflammation → iron dysregulation	
	Low hepcidin (during iron deficiency or increased erythropoiesis) → active FPN allows iron release into blood	In hereditary hemochromatosis: ↓ hepcidin → unregulated FPN → systemic iron overload, oxidative stress → liver fibrosis, cirrhosis, hepatocellular carcinoma.	
Heme/bone marrow	HRG1-mediated intracellular heme transport: HRG1 facilitates heme import into endocytic compartments in the small intestine and regulates intracellular heme distribution (also active in macrophages for iron recycling)		
	Heme synthesis (mitochondrial matrix): Begins with ALAS-catalyzed formation of ALA from glycine and succinyl-CoA (ALAS1 = ubiquitous, ALAS2 = erythroid-specific	ALAS2 mutations/deficiency: Impairs erythroid heme production → sideroblastic anemia ALAS1 regulation disrupted: Toxic heme accumulation in non-erythroid cells	
	Inflammation-mediated inhibition/ACD: Macrophages in erythroblast islands produce itaconate → converted to itaconyl-CoA in erythroid cells → inhibits ALAS2 and reduces succinyl-CoA availability	ACD: Inflammation suppresses heme synthesis via itaconate pathway (dual action: substrate depletion + enzyme inhibition) → reduced hemoglobin synthesis even with sufficient iron stores	

^1^ RX: Therapies. ↑ and ↓ mean increased and decreased respectively.

## Data Availability

All data has been included in the text.
